# Biomarkers in Diabetic Kidney Disease: Early Detection, Prognostic Assessment, and Integration with Multi-Omics Signatures

**DOI:** 10.3390/life16071164

**Published:** 2026-07-14

**Authors:** Merita Rroji, Flaviu Bob, Lorenzo Lo Cicero, Andreja Figurek, Goce Spasovski

**Affiliations:** 1 Faculty of Medicine, University of Medicine Tirana, 1001 Tirana, Albania; 2 Service of Nephrology, University Hospital Center Mother Teresa, 1001 Tirana, Albania; 3 Department of Internal Medicine II—Nephrology, Victor Babes University of Medicine and Pharmacy, Eftimie Murgu Sq. No. 2, 300041 Timisoara, Romania; 4 Centre for Molecular Research in Nephrology and Vascular Disease, Faculty of Medicine, “Victor Babes,” University of Medicine and Pharmacy, 300041 Timisoara, Romania; 5 PhD Program in Molecular and Clinical Medicine, University of Palermo, 90100 Palermo, Italy; 6 Department of Nephrology and Transplantation Medicine, Cantonal Hospital St. Gallen, 9007 St. Gallen, Switzerland; 7 Department of Nephrology, University Ss. Cyril and Methodius, 1000 Skopje, North Macedonia

**Keywords:** diabetic kidney disease, biomarkers, early detection, prognosis

## Abstract

Diabetic kidney disease (DKD) is a leading cause of chronic kidney disease and end-stage kidney disease worldwide, imposing a major clinical and economic burden. Conventional diagnostic markers, including albuminuria and estimated glomerular filtration rate (eGFR), have limited sensitivity and specificity for early disease detection and for accurately predicting progression. Increasing evidence suggests that DKD involves complex glomerular, tubular, inflammatory, fibrotic, and oxidative stress pathways that precede overt clinical manifestations. Consequently, considerable attention has focused on identifying novel noninvasive biomarkers, particularly urinary biomarkers, alongside selected circulating biomarkers and emerging multi-omics signatures. Proteins, peptides, extracellular vesicles, and RNA-based biomarkers have demonstrated promising diagnostic and prognostic potential for detecting early renal injury, improving risk stratification, and monitoring therapeutic response. This review summarizes recent advances in biomarker research for DKD, highlighting emerging molecular and omics-based signatures that may complement conventional markers in improving early detection, prognostic assessment, and disease phenotyping. While numerous biomarkers have shown promising associations with renal outcomes and disease progression, the majority remain investigational. Their translation into routine clinical practice will depend on rigorous external validation, standardized analytical methods, and demonstration of added value beyond established clinical measures.

## 1. Introduction

Diabetic kidney disease (DKD) is the most frequent microvascular complication of type 2 diabetes mellitus (T2D) and a leading cause of end-stage kidney disease (ESKD) worldwide [1,2,3,4]. Despite advances in cardiometabolic care, kidney injury in diabetes remains underrecognized until substantial nephron loss has already occurred. The clinical paradigm still relies on estimated glomerular filtration rate (eGFR) and urinary albumin excretion for the detection and staging of kidney disease [5,6,7]. However, these metrics are imperfect biomarkers of early disease: albuminuria is biologically variable, can be absent in a sizeable subset with progressive “non-albuminuric” NADKD, and often appears after structural injury is established, while eGFR decline lags behind active pathophysiology. Consequently, there is an urgent need for biomarkers that identify kidney injury at its earliest stages, refine risk stratification, and guide timely, mechanism-based interventions [7].

DKD involves interconnected dysfunction of glomerular, tubular, interstitial, and vascular compartments, driven by chronic hyperglycemia, insulin resistance, hemodynamic stress, lipotoxicity, oxidative stress, and sterile inflammation [8,9]. Early events include endothelial glycocalyx damage, podocyte stress and detachment, mesangial expansion, and proximal tubular dysfunction. These changes may be detectable in blood or urine before the onset of persistent albuminuria or a decline in eGFR has occurred [10]. An effective early-detection biomarker should therefore capture one or more of these initiating pathways, exhibit analytical robustness and biological stability, and demonstrate incremental prognostic value beyond albuminuria and eGFR with clear implications on treatment decisions [2,7,10].

A rapidly expanding biomarker toolkit reflects these mechanistic domains, where candidate biomarkers map: glomerular/podocyte injury, tubular stress and injury, inflammatory activation, fibrosis and maladaptive repair, and endothelial dysfunction. In parallel, multi-omics approaches, such as urinary proteomic classifiers, metabolomic and lipidomic fingerprints, and non-coding RNAs in biofluids and extracellular vesicles, offer integrated, non-invasive readouts that may anticipate albuminuria and eGFR decline several years in advance [7].

This review evaluates the current evidence supporting emerging biomarkers for the early detection of DKD, encompassing targeted indicators of glomerular and tubular injury as well as systems-level insights from multi-omics technologies. Additionally, it provides a balanced assessment of mechanistic relevance, diagnostic and prognostic performance, and potential to provide added value beyond traditional clinical indices [11,12], with the aim of advancing biomarker-driven risk stratification, highlighting challenges in standardization and clinical implementation, and supporting the transition toward precision medicine in DKD.

## 2. Methodology

This article presents a narrative review, without quantitative pooling of effect estimates. A structured literature search was conducted by PubMed/MEDLINE, Scopus, and Web of Science for articles published between January 1994 and March 2026. The search strategy combined disease-related terms (such as “diabetic kidney disease,” “diabetic nephropathy,” “chronic kidney disease,” and “type 2 diabetes”) with biomarker- or mechanism-related terms (including “albuminuria,” “estimated glomerular filtration rate,” “tubular injury,” “NGAL,” “KIM-1,” “L-FABP,” “nephrin,” “transferrin,” “MCP-1,” “IL-18,” “TNFR1,” “TNFR2,” “suPAR,” “TGF-β,” “CTGF,” “oxidative stress,” “microRNA,” “proteomics,” “CKD273,” “metabolomics,” “extracellular vesicles,” and “multi-omics”) using Boolean operators. Reference lists of key reviews and primary studies were also hand-searched. Eligible publications included peer-reviewed clinical, observational, translational, and experimental studies, as well as systematic reviews, meta-analyses, and clinical practice guidelines addressing the pathophysiology, diagnostic performance, prognostic value, or clinical application of biomarkers in DKD. Preclinical and mechanistic studies were included only for sections addressing underlying biology, while claims regarding diagnostic or prognostic performance were limited to human cohorts. Conference abstracts without full-text availability, non-English-language publications without accessible translation, and studies lacking sufficient methodological detail to assess validity were excluded. Titles and abstracts were screened for relevance, and full texts of potentially eligible articles were reviewed in detail. Article selection was based on methodological quality, sample size, presence of independent or external validation, and relevance to the mechanistic domains covered in this review (glomerular and podocyte injury, tubular injury, inflammation, fibrosis, oxidative stress, and multi-omics signatures). When multiple studies addressed the same biomarker, preference was given to the larger, multicenter, or multicohort validation studies, and to systematic reviews or meta-analyses over single small studies. To contextualize the strength of evidence supporting each biomarker, a hierarchy of evidence was applied in descending order: (1) systematic reviews and meta-analyses; (2) prospective, multicenter, or multicohort validation studies; (3) single-center prospective cohort studies; (4) retrospective or cross-sectional studies; and (5) mechanistic, preclinical, or exploratory studies providing biological plausibility without direct clinical validation. This hierarchy underpins the evidence-level and clinical-readiness classifications presented in Table 1, Table 2 and Table 3 and informs the qualifying language used throughout the manuscript to describe the clinical maturity of individual biomarkers (such as “established,” “emerging,” “investigational,” or “research-use only”) for implementation in the current clinical practice.

## 3. Pathophysiology of Diabetic Kidney Disease

DKD is increasingly recognized as a heterogeneous, multicompartment disorder involving glomerular, tubular, vascular, and interstitial injury rather than a solely albuminuria-driven condition. Chronic hyperglycemia, insulin resistance, and intrarenal hemodynamic disturbances trigger interconnected metabolic, inflammatory, and oxidative pathways that progressively disrupt renal structure and function [13,14]. Importantly, structural and molecular alterations often precede clinically detectable albuminuria or decline in eGFR, highlighting the limitations of conventional markers for early disease detection.

Endothelial dysfunction, mesangial expansion, podocyte stress, and proximal tubular injury collectively compromise the integrity of the filtration barrier and promote tubulointerstitial remodeling [15,16,17,18,19,20,21,22,23]. The identification of NADKD further demonstrates that tubular and vascular injury can contribute significantly to disease progression independently of overt albuminuria. Persistent inflammatory and profibrotic signaling, along with maladaptive repair responses, ultimately leads to extracellular matrix accumulation and fibrosis, the principal histopathological features of progressive renal decline [24,25,26].

**Figure 1:**
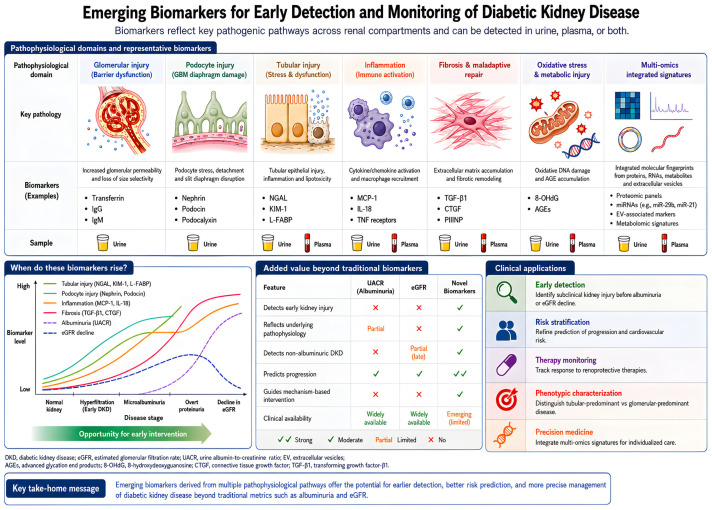
Pathophysiological framework and clinical utility of emerging biomarkers in diabetic kidney disease.

Recent studies implicate epigenetic modifications and persistent metabolic signaling in sustaining inflammatory and profibrotic pathways even as glycemic control improves, contributing to heterogeneity in disease trajectories and clinical phenotypes [27,28]. These pathophysiological mechanisms generate measurable molecular signals in blood and urine that form the basis for biomarker development. Biomarkers reflecting glomerular and podocyte injury, tubular dysfunction, inflammation, fibrosis, oxidative stress, and multi-omics signatures may detect disease activity earlier and more comprehensively than conventional clinical indices [29]. A thorough understanding of these mechanistic domains is essential for interpreting the diagnostic and prognostic value of emerging biomarkers in DKD. Figure 1 provides an overview of the principal pathways and associated biomarker domains.

Emerging biomarkers in diabetic kidney disease (DKD) reflect distinct but interconnected pathophysiological processes across renal compartments, including glomerular, podocyte, tubular, inflammatory, fibrotic, and metabolic pathways. Representative biomarkers include transferrin and immunoglobulins (glomerular permeability), nephrin and podocyte-associated proteins (podocyte injury), NGAL, KIM-1, and L-FABP (tubular stress and injury), MCP-1 and IL-18 (inflammation), TGF-β1 and CTGF (fibrosis), and oxidative stress markers such as 8-OHdG and advanced glycation end products (AGEs). Multi-omics approaches, including proteomic, transcriptomic, and extracellular vesicle–derived signatures, provide integrated molecular profiles of disease activity.

Temporal changes in biomarker expression illustrate that many of these markers are detectable in early or subclinical stages, preceding the onset of persistent albuminuria and decline in estimated glomerular filtration rate (eGFR). Compared with conventional markers, such as urinary albumin-to-creatinine ratio (UACR) and eGFR, these biomarkers offer improved sensitivity for early kidney injury, better characterization of non-albuminuric NADKD, and enhanced mechanistic insight.

## 4. Traditional Biomarkers of Diabetic Kidney Disease

**Albuminuria as a cornerstone biomarker**

Albuminuria, most commonly quantified by the urinary albumin-to-creatinine ratio (UACR), together with eGFR, remains central to the diagnosis, staging, and risk stratification of DKD [30]. Clinically, albuminuria is defined as a UACR ≥ 30 mg/g, corresponding to a urinary albumin excretion of approximately ≥30 mg/day, and is widely recognized as the earliest detectable marker of renal involvement in diabetes. Physiologically, albumin is normally retained within the circulation by the size- and charge-selective properties of the glomerular filtration barrier. In the diabetic milieu, metabolic and hemodynamic perturbations, including insulin dysregulation and glomerular hyperfiltration, disrupt barrier integrity, increasing glomerular permeability and albumin filtration [31].

Although glomerular dysfunction is the primary cause of albuminuria, tubular mechanisms also contribute. Filtered albumin is normally reabsorbed in the proximal tubule via megalin–cubilin-mediated endocytosis, and impaired megalin expression or genetic variants in CUBN increase susceptibility to albuminuria [32,33,34]. Clinically, albuminuria is categorized as A1 (<30 mg/g), A2 (30–300 mg/g), and A3 (>300 mg/g), a staging system that corresponds with disease severity and informs both prognosis and therapeutic decisions [35,36].

This staging system also carries prognostic weight: within the traditional DKD framework, rising albuminuria typically precedes a decline in eGFR and is a strong, independent predictor of progression to ESKD and adverse cardiovascular outcomes [31,37]. Notably, even patients who experience regression of albuminuria continue to exhibit higher residual cardiovascular risk than those who never developed albuminuria [38].

Beyond its role in kidney disease, albuminuria is increasingly acknowledged as a marker of systemic endothelial dysfunction and vascular injury: elevated UACR is independently associated with subclinical atherosclerosis, myocardial infarction, heart failure, fibrosis, and all-cause mortality [33,38,39,40,41]. It is also strongly associated with obesity, metabolic dysfunction-associated steatotic liver disease (MASLD), and insulin resistance, likely reflecting shared mechanisms, including oxidative stress, chronic inflammation, endothelial dysfunction, and intraglomerular hypertension [42,43,44,45,46,47,48,49,50,51,52,53]. Collectively, these associations support the view of albuminuria as an indicator of widespread cardiometabolic stress rather than isolated renal pathology.

This cardiometabolic dimension gives albuminuria direct therapeutic relevance: detecting elevated UACR prompts intensified glycemic and blood pressure management and supports the use of renoprotective and cardioprotective agents, including renin–angiotensin–aldosterone system (RAAS) inhibitors, sodium–glucose cotransporter-2 inhibitors (SGLT2i), nonsteroidal mineralocorticoid receptor antagonists, and glucagon-like peptide-1 receptor agonists (GLP-1 RAs), all of which reduce renal injury and cardiovascular risk [39].

Despite this established clinical utility, albuminuria has important limitations. Its substantial intra-individual variability necessitates repeated measurements, particularly in the A2 range, and its trajectory is not invariably linear: it may regress, remain stable, or progress independently of kidney function decline. Notably, 20–40% of patients with DKD develop NADKD, characterized by impaired eGFR despite normoalbuminuria, likely reflecting predominant tubulointerstitial or vascular injury [31,54,55]. Finally, analytical variability from non-standardized assays may compromise comparability across laboratories, underscoring the need for harmonized reference systems and metrological traceability to enhance the reliability of albuminuria measurement in clinical practice [56].

**eGFR**

Despite its central role in assessing renal function, eGFR typically declines only after significant structural damage has already occurred, thereby limiting its sensitivity for early disease detection [57]. Collectively, these limitations indicate that conventional markers, including albuminuria and UACR, do not fully capture the complexity and heterogeneity of DKD pathophysiology. Such understanding has prompted increased interest in identifying novel biomarkers that reflect complementary dimensions of kidney injury, including glomerular permeability, tubular dysfunction, inflammation, oxidative stress, and endothelial impairment. Compared with conventional markers such as UACR and eGFR, emerging biomarkers offer improved sensitivity and mechanistic insight. Given the marked variability in DKD progression among patients with similar baseline kidney function, early identification of those at high risk is essential for guiding treatment strategies. Table 1 summarizes the clinical advantages of emerging biomarkers compared with traditional markers in diabetic kidney disease.

**Table 1:** Clinical advantages of emerging biomarkers over traditional markers in diabetic kidney disease.

Feature	Albuminuria (UACR)	eGFR	Emerging Biomarkers
Detection of early kidney injury	Limited	Not sensitive	Improved sensitivity
Reflection of pathophysiology	Partial (mainly glomerular)	Functional only	Multi-compartment
Detection of non-albuminuric DKD	Absent	Limited (late detection)	Improved
Sensitivity to dynamic changes	Moderate	Low	Potentially high
Prediction of disease progression	Established	Established	Independent and additive
Risk stratification	Established	Established	Enhanced (multimarker approaches)
Guidance of mechanism-based therapy	Limited	Limited	Emerging potential
Capture of disease heterogeneity	Limited	Limited	Improved
Utility for precision medicine	Minimal	Minimal	Exploratory; not yet clinically actionable
Clinical availability	Widely available	Widely available	Limited

DKD, diabetic kidney disease; eGFR, estimated glomerular filtration rate; UACR, urinary albumin-to-creatinine ratio. Notes: Emerging biomarkers comprise a heterogeneous group of protein-based, inflammatory, fibrotic, oxidative stress–related, and multi-omics markers that reflect complementary pathophysiological processes in DKD. Qualitative descriptors (e.g., “limited”, “moderate”, “improved”, “established”) are derived from the current body of evidence, including observational studies and meta-analyses, and are intended for comparative interpretation rather than as standardized quantitative measures. Conventional markers (UACR and eGFR) remain the foundation of clinical assessment, whereas emerging biomarkers are not intended to replace but to complement these measures by improving early detection, mechanistic insight, and risk stratification. Their clinical utility is currently constrained by limited standardization, variability in assay methodologies, and the need for large-scale validation across diverse populations.

## 5. Emerging Biomarkers in Diabetic Kidney Disease

### 5.1. Protein and Membrane Biomarkers of Glomerular Filtration Barrier Integrity

Among emerging candidates, several protein-based biomarkers have shown promise in detecting early glomerular alterations that precede overt albuminuria. These markers may provide additional sensitivity for identifying subclinical renal injury and offer complementary information to traditional measures.

**Transferrin**

Urinary transferrin (Tf) has emerged as a sensitive biomarker for the early detection and monitoring of DKD. Owing to its relatively low molecular weight (76.5 kDa) and higher isoelectric point, urinary Tf passes earlier than albumin through a compromised glomerular filtration barrier, allowing it to reflect subtle glomerular dysfunction before the onset of overt albuminuria. In patients with T2D, urinary Tf excretion correlates strongly with microalbuminuria but increases to a greater extent, suggesting greater sensitivity to early renal damage [58]. A meta-analysis of six studies reported a pooled sensitivity of 82% (95% CI: 71–89%) and specificity of 88% (95% CI: 84–92%) for urinary Tf in diagnosing early DKD, with a positive likelihood ratio of 7.07, a negative likelihood ratio of 0.20, a diagnostic odds ratio of 34.49, and an AUC of 0.92, indicating excellent discriminative accuracy [59]. As summarized in a review of urinary markers of glomerular injury, urinary transferrin levels are associated with the progression of biopsy-confirmed glomerular lesions and interstitial fibrosis [60], and a separate study reported a progressive rise in urinary transferrin excretion with worsening structural damage [61].

Beyond its diagnostic utility, transferrin may also contribute to disease pathogenesis. Under hyperglycemic conditions, transferrin undergoes non-enzymatic glycation to form advanced glycation end product-modified transferrin (AGE-Tf), which has impaired iron-binding capacity. This directly increases the release of non-transferrin-bound iron, raising oxidative stress through elevated reactive oxygen species (ROS) production. AGE-Tf also induces podocyte injury, apoptosis, and ferroptosis via activation of the receptor for AGEs (RAGE), contributing further to glomerular damage [62].

Overall, these findings support urinary transferrin as a sensitive marker of early glomerular injury with both diagnostic and prognostic value. However, reported diagnostic performance varies considerably across studies, likely reflecting heterogeneity in study populations, diabetes duration, glycemic control, assay methodology, and urine normalization strategies. Urinary transferrin concentrations may also be influenced by hydration status, time of sample collection, recent physical activity, and dietary factors, variables that remain insufficiently standardized across studies. In addition, transferrin excretion is not specific to DKD and may reflect generalized glomerular permeability changes in other kidney diseases [60]. While these limitations persist, ongoing efforts to standardize assays will be essential for broader clinical adoption. These considerations also motivate the search for additional early markers of glomerular injury, such as urinary immunoglobulins, discussed in the following section.

**Urinary immunoglobulins**

In addition to transferrin, urinary immunoglobulin G (IgG) and immunoglobulin M (IgM) provide further insight into early glomerular injury, with their molecular size reflecting barrier integrity. Urinary IgG concentrations increase in diabetes prior to the onset of microalbuminuria and correlate with the severity of glomerular damage [63]. Since IgG is too large to traverse an intact glomerular barrier in significant amounts, its detection indicates a structural defect, and elevated levels are predictive of both DKD onset and progression. Although a threshold of greater than 2.45 mg/L has been proposed to indicate increased risk, this value is derived from a single retrospective cohort and remains provisional [64].

IgM, which is even larger than IgG, is typically absent from urine; therefore, its presence indicates a more substantial breach of the glomerular barrier and is rarely attributable to causes other than significant structural injury. Elevated urinary IgM independently predicts renal function decline regardless of albuminuria status [65]. Additionally, the IgG2/IgG4 ratio provides further detail by detecting early alterations in glomerular size and charge selectivity [66].

These biomarkers provide prognostic information prior to conventional diagnosis. Elevated IgG, particularly in combination with transferrin and ceruloplasmin, predicts the onset of microalbuminuria in normoalbuminuric individuals [67]. A related study observed concurrent increases in IgG, ceruloplasmin, transferrin, and orosomucoid within the same population [68], indicating coordinated, multi-protein glomerular leakage preceding the development of albuminuria. Notably, improved glycemic control reduces urinary immunoglobulin excretion, suggesting that this form of injury may be at least partially reversible [68].

These immunoglobulin markers show that prognostic signal outpaces clinical readiness. They predict outcomes well, including an adjusted hazard ratio of 3.6 for IgM in one cohort [65], yet rest on small, heterogeneous studies and unvalidated thresholds [64], with results further confounded by hydration and concurrent therapy. For now, they should refine risk stratification alongside albuminuria and eGFR, rather than replace them.

**Podocyte-specific biomarkers**

Podocyte injury represents a key early event in the pathogenesis of DKD, reflecting the susceptibility of these highly specialized cells that maintain the glomerular filtration barrier. Under physiological conditions, podocytes form an intricate network of interdigitating foot processes, linked by the slit diaphragm, which facilitates selective filtration of plasma while preventing protein loss [69]. In the diabetic milieu, chronic hyperglycemia triggers a cascade of metabolic and hemodynamic disturbances, including oxidative stress, mitochondrial dysfunction, lipid accumulation, and activation of inflammatory and profibrotic signaling pathways, that compromise podocyte integrity [70]. Multiple intracellular pathways, such as PI3K/Akt, mTOR, JAK/STAT, TGF-β/Smad, Wnt/β-catenin, MAPK, and the NLRP3 inflammasome, have been implicated in this process, collectively promoting podocyte apoptosis, impaired autophagy, and cytoskeletal remodeling [70,71,72,73]. These alterations ultimately disrupt podocyte homeostasis, leading to foot process effacement, detachment, and loss, which clinically manifest as proteinuria.

Among podocyte-associated molecules, nephrin is a central structural and functional part of the slit diaphragm and a key mediator of podocyte integrity. This transmembrane glycoprotein not only supports filtration barrier architecture but also regulates actin cytoskeleton activity and cell survival [74]. In DKD, hyperglycemia-induced oxidative stress, advanced glycation end products, and inflammatory cytokines disrupt the expression, phosphorylation, and localization of nephrin [75]. These changes lead to slit diaphragm disintegration and contribute directly to podocyte injury. Importantly, urinary nephrin excretion (nephrinuria) has emerged as a sensitive and non-invasive biomarker of early glomerular damage, often detectable before the onset of overt proteinuria [76]. Its presence in normoalbuminuric diabetic patients underscores its potential to identify subclinical renal injury earlier than conventional markers.

The clinical relevance of nephrinuria is supported by evidence demonstrating superior diagnostic performance compared with traditional biomarkers, such as microalbuminuria [77,78]. Urinary nephrin levels correlate with established indicators of renal dysfunction, such as albuminuria, serum creatinine, and eGFR, suggesting that nephrinuria reflects both structural and functional impairment [79,80]. Furthermore, reduced nephrin expression in diabetic glomeruli indicates that nephrinuria likely results from dysregulated gene and protein expression rather than passive leakage [33,81,82,83]. In addition to its diagnostic role, nephrinuria appears to have prognostic significance, as elevated levels are linked with disease progression even in the absence of overt proteinuria [84]. However, its clinical implementation remains limited by methodological variability, lack of assay standardization, and the absence of universally accepted reference ranges [33].

Beyond nephrin, several additional podocyte-associated biomarkers offer a complementary perspective on the multifaceted nature of podocyte injury. Slit diaphragm-associated proteins such as podocin and CD2-associated protein (CD2AP) are critical for anchoring nephrin to the actin cytoskeleton, and their urinary expression reflects podocyte stress and detachment. Apical membrane proteins, particularly podocalyxin, help maintain the glomerular charge barrier and are released early during podocyte injury [33]. Notably, intrarenal podocalyxin expression has been associated with adverse renal outcomes, and urinary podocin has shown a modest but significant correlation with the rate of renal function decline [85]. It was reported as a non-invasive indicator of disease progression. These data emphasize the importance of distinguishing between intrarenal and urinary biomarkers when interpreting their clinical significance [86]. Cytoskeletal proteins such as synaptopodin and α-actinin-4 reflect structural changes in podocytes and are linked to disease progression and glomerulosclerosis [86,87]. Emerging biomarkers, including podocyte-derived extracellular vesicles and microparticles, are promising for early detection because they carry proteins and signaling molecules released during cellular stress. RNA-based biomarkers, particularly non-coding RNAs like microRNAs (miRNAs) and long non-coding RNAs (lncRNAs), have also been identified as complementary urinary biomarkers that provide mechanistic insight [88]. These molecules regulate podocyte homeostasis, cytoskeletal organization, and stress-response pathways at the post-transcriptional level. Certain urinary miRNAs, especially those involved in TGF-β signaling, oxidative stress, and apoptosis, display altered expression in early DKD, sometimes before structural or functional abnormalities appear [33]. Unlike protein biomarkers, RNA signatures can reflect upstream regulatory changes and epigenetic reprogramming linked to podocyte dysfunction. However, clinical application is limited by issues such as variable stability in urine, reliance on extracellular vesicle encapsulation, and the absence of standardized normalization methods and reference controls [89]. Additionally, the broad activity and limited cell-type specificity of many miRNAs complicate their interpretation in complex renal diseases. Therefore, while RNA-based biomarkers offer valuable insights into podocyte injury, further standardization and validation are needed before they can be routinely utilized in clinical practice [90].

Recent experimental and clinical studies have provided insight into the therapeutic modulation of podocyte injury. In particular, SGLT2i and hypoxia-inducible factor prolyl hydroxylase inhibitors (HIF-PHI) have been shown to exert direct protective effects on podocytes [91]. Under hyperglycemic conditions, these agents partially restore the expression and localization of key podocyte proteins, including nephrin, podocin, podocalyxin, and synaptopodin, thereby enhancing cytoskeletal organization and cell structure [92]. Observations from kidney biopsy samples further support these data, demonstrating reestablishment of normal podocyte marker distribution following SGLT2i therapy [85,93].

Mechanistically, SGLT2 inhibitors exert pleiotropic effects that counteract the pathogenic processes causing podocyte injury [92]. These include inhibition of mTORC1 signaling, activation of AMPK pathways, attenuation of TGF-β/Smad- and NF-κB-mediated fibrosis, and reduction in oxidative stress by suppressing NOX and NLRP3 inflammasome activity. Through these actions, SGLT2 inhibitors not only reduce glomerular hyperfiltration and proteinuria but also directly preserve podocyte structure and function [92,94,95].

Collectively, podocyte-associated biomarkers reveal a field with one validated leader and a long tail of unproven candidates. Nephrin alone has cleared meaningful evidentiary bars: its association with early glomerular injury has been replicated across independent cohorts, and a recent meta-analysis confirmed high sensitivity, though specificity remained comparatively modest [86]. Podocin, podocalyxin, CD2AP, synaptopodin, extracellular vesicles, and urinary RNA signatures have not cleared this bar; their evidence derives largely from small, single-cohort studies without longitudinal validation [80,85,86,87,88,89]. This distinction matters because consistent correlation with albuminuria and eGFR is not the same claim as added predictive value, and at present none of these markers, nephrin included, has demonstrated that it improves risk prediction beyond what albuminuria and eGFR already provide. Part of this uncertainty is methodological rather than biological: assay platforms, urine normalization, and sampling conditions vary across studies, and the effects of glycemic control and nephroprotective therapies such as SGLT2 inhibitors on these markers remain largely unexamined. Until that methodological noise is resolved through standardized, prospective validation in ethnically diverse cohorts, the central question for podocyte biomarkers, whether they add anything to what clinicians can already measure, remains open.

### 5.2. Tubular Injury Biomarkers: Indicators of Early Tubulointerstitial Damage

Tubulointerstitial injury is increasingly recognized as a central component in the pathogenesis of DKD, often manifesting before clinically evident glomerular dysfunction or measurable decline in renal function. Conventional biomarkers, particularly albuminuria, primarily reflect glomerular damage and may not adequately capture early tubular involvement. This limitation has driven growing interest in biomarkers that more specifically reflect tubular injury, among which neutrophil gelatinase-associated lipocalin (NGAL) has emerged as one of the most extensively investigated candidates [96].

**NGAL**

NGAL is a low-molecular-weight protein initially identified in neutrophils but subsequently recognized as a stress-responsive molecule markedly upregulated in renal tubular epithelial cells following ischemic, inflammatory, or toxic injury. Its rapid induction and release into both plasma and urine render it a sensitive indicator of early tubular stress, often detectable before alterations in eGFR or the onset of albuminuria. This early responsiveness positions NGAL as a valuable marker for identifying subclinical renal injury in patients with diabetes [97,98,99].

Clinical studies have consistently demonstrated that elevated NGAL levels are associated with key indicators of disease severity, including reduced eGFR, increased albuminuria, and longer duration of diabetes. Notably, differential patterns between circulating and urinary NGAL have been described. Plasma NGAL concentrations may increase in individuals with longstanding diabetes even in the absence of overt kidney disease, whereas urinary NGAL elevations are more closely linked to established renal involvement, particularly in the presence of significant albuminuria [100]. These observations suggest that NGAL reflects both systemic and kidney-specific processes, depending on the biological compartment assessed.

Meta-analytic data further support the diagnostic utility of NGAL, with reported high sensitivity and specificity, particularly in longitudinal cohort studies. However, variability across study designs and patient populations highlights the need for cautious interpretation and standardization of cutoff values [101]. In clinical practice, urinary NGAL is frequently normalized to creatinine (uNGAL-to-creatinine ratio), which has been shown to correlate with histopathological evidence of tubulointerstitial damage, proteinuria, and declining renal function [102]. Elevated values have also demonstrated potential utility in distinguishing DKD from non-diabetic renal pathology and may assist in guiding decisions regarding kidney biopsy [103].

Despite these promising findings, NGAL performance appears to be influenced by disease stage [99]. While consistently elevated in advanced DKD and associated with disease progression, its predictive capacity in early-stage disease remains less consistent. In cohorts with minimal tubular injury, NGAL may lack sufficient sensitivity to independently predict future decline in renal function. This stage-dependent behavior underscores the importance of integrating NGAL within multimarker strategies rather than relying on it as a standalone indicator [99].

Beyond its role as a biomarker, NGAL is also implicated in the pathophysiology of renal injury. It participates in processes such as modulation of tubular cell proliferation, apoptosis, and inflammatory signaling, including pathways involving epidermal growth factor receptor activation and hypoxia-inducible factors. These mechanistic associations further support its relevance as both a marker and mediator of the disease progression [104,105,106].

Prospective studies have demonstrated that elevated baseline urinary NGAL levels are associated with a higher risk of rapid decline in kidney function and rapid progression to ESKD, independent of conventional risk factors. Collectively, these findings highlight the dual diagnostic and prognostic utility of NGAL [99]. Its non-invasive measurement and ability to capture early tubular injury provide meaningful advantages over traditional markers, although variability in assay methodologies and thresholds continues to limit its routine clinical implementation [107].

Among tubular injury biomarkers, NGAL has the most robust evidence base in DKD, with consistent associations with albuminuria, declining eGFR, and adverse renal outcomes across observational studies, prospective cohorts, and meta-analyses [98,99,100,101,103,104,105]. However, this strength is primarily observed in a specific context: NGAL performs optimally as a marker of established or progressive disease, whereas its predictive value in normoalbuminuric or very early DKD remains inconsistent [99,101,105]. Furthermore, the diagnostic utility of NGAL is influenced by the measured biological compartment; urinary NGAL provides greater kidney specificity than plasma NGAL, which is affected by systemic inflammation and comorbidity burden [100,101]. This compartment and stage-dependence represent a central limitation, indicating that NGAL’s clinical value is conditional rather than universal. The substantial variability in reported performance across studies likely reflects this dependence as much as differences in assay methodology. Consequently, NGAL is closer to clinical translation than most tubular biomarkers, but current evidence supports its use as a complementary marker of tubular stress and disease progression rather than as a standalone diagnostic test.

**KIM-1**

Beyond NGAL, kidney injury molecule-1 (KIM-1) has emerged as a highly informative biomarker of tubular damage in DKD, particularly in the early phases preceding the onset of clinically detectable albuminuria. Under physiological conditions, KIM-1 expression in the kidney is minimal, while it is rapidly induced in proximal tubular epithelial cells following injury. The extracellular domain of KIM-1 is subsequently cleaved and released into the urine, enabling non-invasive assessment of tubular epithelial stress and damage [102,108].

Accumulating evidence indicates that tubular dysfunction represents an early and independent component of DKD pathogenesis. In this context, elevated urinary KIM-1 (uKIM-1) levels have been consistently detected in patients with T2D even in the absence of albuminuria, supporting its role as a marker of subclinical renal injury [109,110]. Associations with metabolic disturbances, including insulin resistance, further suggest that KIM-1 reflects early pathogenic processes not captured by traditional glomerular markers. These findings underscore the limitations of albuminuria as a sole indicator of kidney involvement and reinforce the contribution of tubulointerstitial injury to the disease initiation [111,112].

As DKD progresses, uKIM-1 concentrations show robust associations with established markers of renal impairment. Positive correlations have been reported with proteinuria, serum creatinine, and markers of inflammation and fibrosis, alongside an inverse association with eGFR. Interestingly, some studies suggest that uKIM-1 levels may peak during earlier stages of CKD, potentially reflecting active tubular injury, whereas lower levels in advanced stages may be attributable to nephron loss and reduced cellular mass [109,110,113]. This stage-dependent pattern highlights the dynamic nature of KIM-1 expression across the disease continuum.

From a prognostic perspective, elevated uKIM-1 levels have been linked with adverse renal outcomes and increased mortality in patients with diabetes and CKD, independent of conventional risk factors [114]. However, the strength of these associations varies across studies, with some analyses reporting attenuation after adjustment for clinical covariates, indicating that their predictive value may be influenced by population characteristics and study design [115]. When combined with β2-microglobulin and standard clinical variables such as eGFR and albuminuria, it provided a modest but reproducible improvement in risk prediction across diverse cohorts. Interestingly, larger biomarker panels do not enhance predictive performance beyond this combination, highlighting KIM-1 as a key, nonredundant biomarker for stratifying risk of kidney disease progression [116,117]. These discrepancies emphasize the need for standardized assessment frameworks and validation in larger, diverse cohorts [33].

Beyond its utility as a biomarker, KIM-1 is increasingly identified as an active participant in the disease pathophysiology. In acute injury, KIM-1 contributes to epithelial repair through the phagocytic clearance of cellular debris. In contrast, sustained expression in chronic conditions may promote maladaptive responses, including persistent inflammation, oxidative stress, and fibrogenesis. Mechanistically, KIM-1 has been implicated in mediating the uptake of albumin-bound lipids by proximal tubular cells, thereby amplifying lipotoxicity and downstream inflammatory signaling pathways. These effects may contribute to progressive tubulointerstitial damage and secondary glomerulosclerosis. Notably, experimental inhibition of KIM-1-mediated pathways has been associated with attenuation of renal injury, supporting its potential as a therapeutic target [33,108,118,119].

Current evidence suggests that KIM-1 is supported by strong biological plausibility and an expanding body of observational and prospective evidence linking elevated levels with renal function decline, CKD progression, and mortality in DKD [109,110,111,112,113,114,115,116,117,118,119]. Nevertheless, the strength of these associations is not entirely consistent, with several studies reporting attenuation after adjustment for established clinical predictors, including albuminuria, eGFR, and metabolic risk factors [114,115,116,117]. These discrepancies may partly reflect differences in disease stage, as KIM-1 may be most informative during periods of active tubular epithelial injury, whereas lower concentrations in advanced disease may result from nephron loss and reduced tubular cellular mass [109,110,113]. Variability in assay methodology, study design, follow-up duration, and population characteristics may further contribute to heterogeneous findings. Consequently, KIM-1 currently appears most clinically valuable as an adjunctive biomarker for risk stratification within multimarker approaches rather than as an independent replacement for conventional measures [114,115,116,117].

**L-FABP**

In addition to KIM-1, liver-type fatty acid-binding protein (L-FABP) provides complementary insight into the metabolic and lipid-mediated stress within proximal tubular cells, capturing a distinct dimension of tubular injury in DKD. L-FABP is a small cytosolic protein predominantly expressed in renal proximal tubular epithelial cells, where it facilitates intracellular transport and metabolism of free fatty acids [120]. Under conditions of metabolic stress, including hyperglycemia and increased lipid exposure, its expression is upregulated and subsequently released into the urine, enabling non-invasive assessment of tubular dysfunction [120,121,122]. Mechanistically, increased urinary L-FABP reflects enhanced tubular exposure to free fatty acids and relative hypoxic stress, both of which contribute to oxidative injury and progressive tubulointerstitial damage.

Clinical evidence indicates that urinary L-FABP (uL-FABP) levels are elevated in patients with diabetes even in the absence of albuminuria, suggesting that tubular injury may occur early in the disease course, prior to detectable glomerular involvement [121]. Furthermore, uL-FABP concentrations increase progressively with advancing disease severity, demonstrating positive associations with albumin excretion and inverse relationships with eGFR, thereby reflecting both structural and functional deterioration [120,121,122].

Beyond its diagnostic role, uL-FABP has also demonstrated a significant prognostic value. Longitudinal studies show that elevated baseline levels are associated with an expanded risk of developing microalbuminuria, progression to advanced nephropathy, accelerated decline in renal function, and progression to ESKD [121,123,124]. In addition, higher uL-FABP concentrations have been linked to an increased cardiovascular risk, underscoring its broader relevance as a marker of systemic metabolic and vascular stress [125].

From a practical perspective, uL-FABP offers several advantages that support its clinical applicability. It can be reliably measured in urine using standardized assays, including enzyme-linked immunosorbent techniques and point-of-care platforms, facilitating its use in both specialized and resource-limited settings [126]. Moreover, dynamic changes in uL-FABP levels have been shown to reflect response to renoprotective and glucose-lowering therapies, suggesting a potential role in monitoring treatment efficacy and guiding clinical decision-making [122,126,127].

Importantly, uL-FABP provides complementary information to traditional biomarkers. While albuminuria predominantly reflects glomerular injury, L-FABP captures tubular-specific processes, allowing a more integrated assessment of renal involvement in diabetes. The combined use of these markers may enhance diagnostic accuracy and improve risk stratification by capturing distinct but interrelated aspects of kidney pathology [126,128].

Compared with NGAL and KIM-1, evidence supporting L-FABP is promising but comparatively less mature. Available longitudinal studies and meta-analytic data support associations between elevated urinary L-FABP and nephropathy progression, accelerated eGFR decline, and cardiovascular risk [121,123,124,125]. Its principal advantage lies in its mechanistic specificity for lipid-mediated tubular stress and hypoxic injury, thereby capturing aspects of DKD pathophysiology that are not adequately reflected by glomerular biomarkers alone [120,121,122]. However, replication in large and ethnically diverse populations remains limited, and the magnitude of its incremental predictive value beyond albuminuria and eGFR has not yet been consistently established [121,124,125]. Therefore, while L-FABP represents a promising component of multimarker strategies, further validation and harmonization of assays are needed before routine clinical implementation can be proposed [128].

Taken together, tubular injury biomarkers provide compelling evidence that DKD extends beyond glomerular dysfunction and involves early tubulointerstitial injury that may precede overt albuminuria. NGAL and KIM-1 currently appear the most clinically mature biomarkers because their associations with renal decline and adverse outcomes have been replicated across multiple observational and prospective studies [101,114,115,116,117]. By contrast, L-FABP offers valuable mechanistic insight into lipid-mediated tubular stress and hypoxic injury but remains comparatively less validated in large external cohorts [121,123,124,125,126]. At present, these biomarkers should be regarded as complementary rather than substitutive tools, adding pathophysiological and prognostic information to established measures such as UACR and eGFR.

Clinical translation, however, remains constrained by several unresolved issues, including assay heterogeneity, lack of universally accepted thresholds, and uncertainty regarding the influence of hydration status, physical activity, diet, and treatment exposure on urinary biomarker concentrations [101,116,121,126]. Moreover, whether biomarker dynamics primarily reflect active disease, irreversible structural injury, or therapeutic response remains incompletely understood. Accordingly, the most realistic near-term application lies in multimarker models that integrate tubular biomarkers with conventional clinical variables to improve risk stratification and earlier identification of progressive DKD.

Beyond these tubular injury markers, a further group of biomarkers reflects the inflammatory and oxidative pathways that drive disease progression in DKD, discussed below.

### 5.3. Markers of Inflammation and Oxidative Stress

**MCP-1/CCL2**

A marker of the progression of DKD is represented by the infiltration of monocytes and macrophages in the kidneys. The accumulation of macrophages at that level is influenced by MCP 1, a member of the CC chemokine family. The level of urinary MCP-1 correlates with metabolic and inflammatory stress, suggested by the correlation with hyperglycemia (HbA1c) and inflammatory markers (IL-6, TNF-α), as it was shown in a cross-sectional study performed in 185 patients [129].

Regarding the relationship with DKD, MCP-1 seems to be a marker, and also a mediator of an early diabetic nephropathy. In a case control study including patients of the Randomized Olmesartan And Diabetes Microalbuminuria Prevention (ROADMAP) study and its Observational Follow-up (OFU) cohort, it has been shown that serum and urinary MCP-1 predicted the development of the early biomarker microalbuminuria, suggesting MCP-1 elevation precedes clinical DKD [130]. Regarding the relationship of urinary MCP-1 with albuminuria in DKD patients, in a meta-analysis, five case control studies, comprising 295 patients, have been identified and the correlation coefficient was 0.694 (95% CI: 0.575–0.784, *p* < 0.001) [131].

Besides being an early marker of DKD, urinary MCP-1 shows also the degree of kidney disease progression, varying according to different albuminuria statuses and eGFR in diabetic patients. Regarding the association with outcomes of DKD, prospective observational studies have shown that urinary MCP-1 was positively associated with the risk of doubling of serum creatinine or death [105,132,133].

The pathological link between the upregulation of MCP-1 and DKD is represented by the fact that the expression of MCP-1 in renal cells is induced by inflammatory cytokines and constitutes the starting point for the development of glomerular and tubular inflammation [134]. The extent of interstitial inflammatory infiltrate correlates with the expression of MCP-1 in the diabetic glomerular and tubular epithelium [22].

There is also a direct effect of the diabetic milieu on MCP-1 production in mesangial cells or tubular epithelial cells, via NF-kB, ROS or other pathways. On the other hand, MCP-1 leads to increased expression of fibronectin and type IV collagen in mesangial cells, suggesting that MCP-1, which is produced by mesangial cells in hyperglycemic conditions, may also play an autocrine role in extracellular matrix deposition, as it has been shown in studies performed with cell cultures [135].

The evidence, although deriving mainly from small case-control studies, indicates that MCP-1 is not merely a marker but likely a mediator in the pathogenesis of DKD. MCP-1 is upregulated in the diabetic kidney, correlates with worse disease, promotes macrophage recruitment and inflammation, and genetic/therapeutic inhibition of the MCP-1 pathway mitigates renal injury in animal models.

**IL-18**

Besides MCP-1, different inflammatory mediators have an important role in the pathophysiology of DKD, such as different members of the interleukin family. A specific role is played by IL-18, expressed at the level of the renal structures and upregulated by different stimuli, such as hyperglycemia, but also hyperlipemia, oxidative stress or hyperuricemia [136]. The link between metabolic abnormalities and interleukin production is through an activation of different inflammasomes, such as NLRP3 [33]. In order to be activated, inactive IL-18 must be cleaved by caspase-1, afterwards it induces inflammatory mediators through the NF-kB pathway [137]. Activation of IL-18, together with other different cytokines and chemokines (IL-1beta, TNF alpha, IL6, etc.) lead to podocyte damage, mesangial cells accumulation, extracellular matrix synthesis and albuminuria. On the other hand, in an animal model of DKD, the inhibition of NLRP3 and caspase-1 protects the subject from diabetic-mediated renal damage [138].

Both the expression and urinary level of IL-18 correlate with the disease progression. Tubular cells are not only origin, but are also target of IL-18. The pathway represented by NLRP3 and IL-18 is not only responsible for the pyroptotic cell death in AKI, but is involved in CKD, including DKD, by the promotion of fibrosis [139].

Therefore IL-18 could be used as a biomarker of renal involvement in diabetic patients. In a cross-sectional study performed in 250 patients and 65 healthy controls higher IL-18 concentrations in patients with more advanced diabetic kidney disease have been reported [140]. Higher levels of IL-18 could also be predictive of future renal dysfunction in type 2 diabetic patients with normoalbuminuria in an observational study performed in 249 patients followed up for 7 years [141].

Besides being central to T2D pathophysiology, inflammation is also linked to CV disease. The connection between renal inflammation and systemic vascular damage is proven by the fact that urinary IL-18 is associated to increased arterial stiffness in patients with DKD, as it has been shown in 180 patients with and without renal disease [142].

The knowledge of the role of IL18 could indicate a potential therapeutic role of targeting IL-18 or upstream inflammasome pathways (especially NLRP3) in DKD. Recent animal studies suggest that SGLT2 inhibitors modulate the NLRP3 inflammasome and thus reduce pro-inflammatory markers [143]. Thus, the role of IL-18 as a marker of progression of DKD has been proven in large but observational studies.

**TNFR1 and TNFR2**

Tumor necrosis factor receptors 1 and 2 (TNFR1 and TNFR2) have emerged as robust inflammatory and prognostic biomarkers in DKD, reflecting activation of tumor necrosis factor (TNF)-mediated inflammatory pathways implicated in progressive renal injury [144,145,146]. Elevated circulating TNFR concentrations have been consistently associated with accelerated decline in kidney function, progression to ESKD, and mortality, independent of conventional renal risk markers such as albuminuria and eGFR [144,145,146]. In a landmark prospective study by Niewczas et al., involving patients with T2D followed for 12 years, individuals within the highest quartile of circulating TNFR1 demonstrated a cumulative incidence of ESKD of approximately 54%, compared with only 3% among those in the lower quartiles, highlighting the remarkable prognostic strength of these biomarkers [144]. Importantly, the predictive value of TNFR1 and TNFR2 remained significant after adjustment for baseline renal function and albuminuria, suggesting their ability to identify high-risk individuals before clinically advanced DKD becomes evident [144,145].

Subsequent studies reinforced the role of TNFRs as early indicators of DKD progression. Gohda et al. demonstrated that circulating TNFR1 and TNFR2 concentrations were significantly elevated in patients with type 2 diabetes despite preserved kidney function, with median TNFR1 and TNFR2 concentrations of 1357 pg/mL and 2904 pg/mL, respectively, compared with 992 pg/mL and 1951 pg/mL in healthy controls (*p* < 0.0001 for both) [147]. Furthermore, TNFR concentrations correlated inversely with cystatin C–based eGFR (TNFR1: r = −0.30; TNFR2: r = −0.28; *p* < 0.001), supporting their potential utility as biomarkers of early renal dysfunction before overt kidney impairment becomes clinically apparent [147]. Beyond functional decline, elevated TNFR levels have also been associated with structural kidney injury, including mesangial expansion, glomerular endothelial damage, and tubulointerstitial fibrosis, suggesting that these biomarkers may reflect ongoing renal tissue remodeling rather than systemic inflammation alone [148].

The prognostic significance of TNFR1 and TNFR2 has been further substantiated by meta-analytic evidence. A 2019 meta-analysis demonstrated that individuals with the highest circulating TNFR concentrations had a significantly increased risk of DKD progression compared with those with lower levels, with pooled risk ratios of 2.51 (95% CI: 1.92–3.27) for TNFR1 and 3.23 (95% CI: 1.99–5.26) for TNFR2 [149]. These findings reinforce the reproducibility and robustness of TNFRs as prognostic biomarkers across diverse diabetic populations. Importantly, recent therapeutic studies suggest that TNFR1 and TNFR2 may also function as biomarkers of treatment response. In the CANVAS trial, treatment with the SGLT2-i canagliflozin modestly reduced circulating TNFR1 and TNFR2 concentrations compared with placebo, while early reductions in TNFR levels were independently associated with lower subsequent kidney risk [150]. These observations suggest that TNFR1 and TNFR2 may not only identify patients at increased risk of DKD progression but also reflect modulation of inflammatory pathways in response to renoprotective therapies. Nevertheless, despite their strong predictive performance, further studies are required to establish standardized thresholds and define their integration into routine nephrology practice.

**suPAR**

Soluble urokinase plasminogen activator receptor (suPAR) has emerged as a promising biomarker for renal injury and disease progression in DKD, reflecting the complex interactions among chronic inflammation, innate immune activation, and structural kidney damage [151,152]. Elevated circulating suPAR concentrations are consistently associated with worsening renal outcomes, indicating potential utility beyond conventional markers such as albuminuria and eGFR [151,153].

Clinical studies demonstrate a strong association between suPAR levels and the severity of renal dysfunction in DKD. In a cross-sectional study by Lupușoru et al. involving 75 patients with DKD, median serum suPAR concentrations were 2857.2 pg/mL in the overall cohort and increased significantly as kidney function declined, from 1610.1 pg/mL in early chronic kidney disease (CKD) stages to 3236.1 pg/mL in advanced disease (CKD G3b–G5; *p* < 0.001) [152]. Serum suPAR levels correlated inversely with eGFR (r = −0.684, *p* < 0.001) and positively with albuminuria (r = 0.525, *p* < 0.001) and 24-h proteinuria (r = 0.490, *p* < 0.001), supporting its association with progressive renal impairment [152].

In addition to functional decline, suPAR is associated with histopathological severity in biopsy-confirmed DKD. Higher suPAR concentrations are observed in advanced Renal Pathology Society (RPS) classes, reaching 3940.7 pg/mL in class III disease compared with 1491 pg/mL in class I nephropathy [152]. Similarly, serum suPAR increases progressively with worsening interstitial fibrosis and tubular atrophy (IFTA), rising from 1738 pg/mL in mild lesions to 3272.7 pg/mL in severe tubulointerstitial injury (*p* = 0.02). Significant correlations are also reported between suPAR levels and histological class (r = 0.493, *p* = 0.008), IFTA score (r = 0.506, *p* = 0.006), and interstitial inflammation (r = 0.412, *p* = 0.029), indicating that suPAR reflects both glomerular and tubulointerstitial injury [152].

The prognostic value of suPAR extends beyond disease characterization to early risk stratification. Hayek et al. demonstrated that elevated suPAR concentrations independently predict kidney function decline. Individuals in the highest quartile experienced a substantially greater annual reduction in eGFR compared to those in the lowest quartile (−4.2 vs. −0.9 mL/min/1.73 m^2^/year) and had a 3.13-fold increased risk of progression to CKD stage 3, independent of conventional risk factors [151]. Similarly, Guthoff et al. reported that elevated suPAR levels predicted the onset of microalbuminuria before the development of clinically overt DKD [154].

In addition to its prognostic role, emerging evidence indicates that suPAR may actively contribute to the pathogenesis of DKD rather than solely reflect renal injury. Mechanistically, suPAR promotes activation of αvβ3 integrin signaling in podocytes, leading to cytoskeletal rearrangement, foot process effacement, and disruption of the glomerular filtration barrier, which contributes to proteinuria [155]. Recent studies suggest that suPAR acts synergistically with other nephrotoxic factors, including chronic inflammation, metabolic stress, and APOL1 risk variants, rather than inducing kidney injury independently [156,157]. This perspective positions suPAR within the broader innate immunity and kidney axis and may partly explain interindividual variability in DKD progression [156]. Additionally, suPAR demonstrates relative long-term stability and minimal circadian variability, enhancing its suitability as a biomarker for chronic renal risk assessment [156]. Emerging anti-suPAR therapeutic strategies, including monoclonal antibodies under clinical investigation, further support the potential of suPAR as a biomarker-guided therapeutic target in DKD [156]. However, larger prospective studies are necessary before routine clinical implementation can be recommended [151,156].

### 5.4. Markers of Fibrosis and Extracellular Matrix (ECM) Remodeling

**TGF-β**

TGF-β1 is the most frequent of the isoforms of TGF and is synthesized by all types of resident renal cells and infiltrating inflammatory cells. The activation of the latent form of TGF-β1 occurs due to hyperglycemia, mechanical stretch, increased angiotensin II or advanced glycation end products. Active TGF-β1 interacts with its receptors and leads to downstream signaling through Smad-dependent and Smad-independent pathways [158].

The result of TGF-β1 activation is involvement in fibrogenesis, and the mechanism is multifactorial, involving increased production and decreased degradation of extracellular matrix, but also activation of the de-differentiation of proximal tubular and endothelial cells, leading to interstitial fibrosis. In addition, TGF-β1 is exerting its effect on podocytes throughout foot processes effacement with consequent increased glomerular permeability, but also reduction in podocyte migration, resulting in podocyte death and glomerulosclerosis [159].

Further evidence supporting the involvement of TGF-β1 in the pathogenesis of DKD comes from renal biopsy studies, which have demonstrated increased TGF-β1 expression in areas of glomerulosclerosis and interstitial fibrosis [8].

Measuring the level of urinary or serum TGF-β1 could be helpful as marker of DKD, with levels being higher in patients compared to controls as it has been confirmed in different case-control studies with subject numbers ranging from 20 to below 130. It has also been shown that higher blood levels of TGF-β could be associated with early stages of DKD, and that there is a positive correlation between levels in the urine and sera with proteinuria in these patients [160,161].

At present, there are some available data with regard to the therapeutic efficacy of blocking TGF-β. Surprisingly, when studied in diabetic animals, the use of anti-TGF-β antibodies induced a reduction in glomerular hypertrophy, the ECM accumulation and renal fibrosis, without influencing albuminuria [162].

In human clinical trials, it has been observed that baricitinib, an oral, reversible, selective inhibitor of Janus kinase 1 (JAK1) and JAK2, and fenofibrate both lead to a reduction in albuminuria in diabetic patients.

On the other hand, some interventions show that modulating TGF-β, rather than directly blocking TGF-β ligands/receptors, may be a good antifibrosis strategy, because throughout a complete blockade, the beneficial functions of TGF-β are blocked as well [161]. TGF-β functions both as a marker of DKD, as has been proven in observational studies, and as a treatment target, as shown not only in animal studies, but also in small trials.

**CTGF**

A key downstream mediator of renal fibrosis induced by TGF-β is represented by Connective Tissue Growth Factor (CTGF). Increased CTGF expression in renal cells in DKD is induced by several different factors, including the high glucose environment, mechanical strain, TGF-β, AGEs proteins and ROS [163].

CTGF increases synthesis of fibronectin and plasminogen-activator inhibitor-1 [164]. At the level of the renal tissue CTGF expression is mainly localized in podocytes and parietal glomerular epithelial cells, and less prominent in mesangial cells [165].

The result of CTGF stimulation is represented by extracellular matrix accumulation and fibrosis [166]. It could be considered that CTGF is involved in the progression of DKD, being also a biomarker of the severity of the disease.

In a large cross-sectional study of 318 patients with type 1 diabetes it has been shown that the urinary level of CTGF is correlating with albuminuria and eGFR [167], whereas plasma CTGF showed a correlation with mortality and progression to ESKD in a study performed in 386 patients followed up for 12.8 years [168].

Antagonizing CTGF function leads to a reduction in the progression of glomerulosclerosis, leading to a non-complete blockade; this fact seems to avoid the dangers associated with the blockade of TGF-β. The use of a human monoclonal antibody on CTGF in a phase 1 study of DKD patients was well tolerated and associated with a decrease in albuminuria [169].

Although large-cohort studies and one clinical trial support the role of CTGF (urinary and plasmatic) both as disease marker and as treatment target, the current evidence base remains limited, and additional validation studies are needed before CTGF can be considered a clinically established biomarker. 

**Collagen IV and fibronectin**

As already mentioned above, the hallmark of renal fibrosis is extracellular matrix production and deposition. Of the key ECM proteins, collagen IV and fibronectin are associated with the development and progression of DKD, being involved in the mesangial expansion, thickening of the GBM, glomerulosclerosis and tubulointerstitial fibrosis [170].

The expression of these proteins in DKD kidneys is altered. Both, fibronectin-1 and collagen IV are certainly present in the healthy human kidney. Type IV collagen is an abundant protein of the glomerular ECM and may be observed in the GBM and the mesangium [170].

The composition and arrangement of the glomerular ECM is profoundly altered in patients with diabetes. Immunohistochemical studies reveal that the amount of mesangial fibronectin-1 is abnormal and varies with the DKD progression, while the type IV collagen in the GBM shows reduced staining compared to normal tissue [171].

Both ECM proteins have adopted their role as biomarkers of DKD. Urinary type IV collagen is an indicator of early onset and disease progression in both type-1 and type-2 diabetic patients, and its higher concentration in the serum indicates the progression of kidney disease, as it has been shown in a multicenter study performed in 889 patients [172]. However, these results have not been confirmed in other studies [173]. Urinary and plasma fibronectin was found to be linked with micro- and macrovascular complications such as nephropathy, retinopathy, neuropathy, and cardiovascular incidence among diabetic patients, but due to the fact that fibronectin can be synthesized from different sources from the kidney, its role as a marker of DKD still needs to be established [174].

### 5.5. Markers of Oxidative and Metabolic Stress

**8-OHdG**

In the pathogenesis of DKD, besides metabolic control and hemodynamic alterations, there is also involvement of oxidative stress. Here, DNA oxidation is represented by 8-hydroxy-2′-deoxyguanosine (8-OHdG) marker.

The fact that 8-OHdG is found in plasma and is excreted in urine after DNA repair by nuclease activity suggests that it could be used as a biomarker of oxidative DNA damage in diabetic patients [175]. Serum and urine levels of 8-OHdG are increased with the degree of albuminuria, and indirectly correlate with antioxidant markers, such as superoxide dismutase (SOD) [176]. It has been also shown that 8-OHdG plasma levels are associated with an increased risk of kidney disease in individuals with type 1 diabetes as it has been shown in participants from two large cohorts GENEDIAB (*n* = 348) and GENESIS (*n* = 571) [177].

The proof of the involvement of oxidative stress in DKD comes also from renal biopsy studies, which show increased 8-OHdG staining in the glomeruli, indicating local oxidative DNA damage in the kidneys [176].

While large cohort studies support an association between 8-OHdG and DKD progression, additional studies are needed to better define the diagnostic and prognostic value of oxidative stress biomarkers in DKD. 

**Advanced glycation end products**

Hyperglycemia-related AGEs formation plays a central role in the pathogenesis of DKD. The accumulation of RAGE triggers oxidative stress and inflammation [177,178].

The severity of the expression in glomerular and tubulointerstitial compartments of AGE and RAGE correlates with DKD. The presence of AGEs alters the metabolism of ECM components leading to a thickening of the glomerular and tubular basement membranes. AGEs are also involved in the podocyte dysfunction, as well as in the macrophage migration and inflammation [179].

It was shown in a study of nondiabetic rats in whom AGEs were administered that proteinuria was obtained, this being proof of the role of AGEs in the pathogenesis of renal damage [180].

In large cohorts of subjects with T2D (Action to Control Cardiovascular Risk in Diabetes -ACCORD, *n* = 1150 and Veterans Affairs Diabetes Trial-VADT-*n* = 447), increased levels of AGEs, assessed using composite AGE scores (multiple AGE metabolites), were associated with the progression of DKD, showing the potential role of AGEs as markers of DKD [181].

Targeting RAGE with its ligands mediated oxidative stress and chronic inflammation is considered as an additional intervention strategy for DKD. The adoption of an anti-AGE therapy could be a good choice for possible DKD prevention. The regimen should regulate blood glucose to the normal range, use antioxidants and anti-AGE compounds in the diet to protect from AGE-mediated complications and improve renal function, particularly in diabetics [182]. Table 2 summarizes the clinical utility of emerging biomarkers in diabetic kidney disease, including their role in early detection, risk stratification, and prediction of disease progression and key recommendations for clinicians are presented in Box 1.

**Table 2:** Clinical readiness of emerging biomarkers in diabetic kidney disease: diagnostic, prognostic, and translational implications.

Biomarker	Pathway	Sample	Stage Detected	Main Clinical Utility	Evidence Level	Incremental Value Beyond UACR/eGFR	Clinical Readiness	Major Limitation
**Transferrin**	Glomerular permeability	Urine	Pre-albuminuria	Early detection of glomerular injury; prediction of incident microalbuminuria	Meta-analysis and longitudinal studies	Uncertain	Emerging clinical candidate	Limited specificity and lack of assay standardization
**IgG/IgM**	Glomerular barrier disruption	Urine	Early–Advanced	Reflect glomerular permeability changes and predict progression	Cohort studies	Limited	Experimental	High biological variability and absence of validated cut-offs
**Nephrin**	Podocyte injury	Urine	Very early	Detection of podocyte injury before overt proteinuria	Cohort studies and systematic review/meta-analysis	Uncertain	Emerging clinical candidate	Assay variability and lack of reference ranges
**Podocin, Podocalyxin, CD2AP, Synaptopodin**	Podocyte injury	Urine	Very early	Detection of podocyte stress and detachment	Small observational studies	Not established	Research-use only	Insufficient longitudinal validation
**NGAL**	Tubular injury	Urine/Plasma	Early–Advanced	Detection of tubular injury; prediction of eGFR decline and DKD progression	Meta-analyses and prospective cohorts	Moderate	Advanced clinical validation	Assay heterogeneity and variable performance in very early disease
**KIM-1**	Tubular injury	Urine/Plasma	Early	Detection of proximal tubular injury and improved risk stratification	Multiple cohorts and prospective studies	Moderate	Advanced clinical validation	Lack of standardized thresholds and variable effect sizes
**L-FABP**	Tubular stress/hypoxia	Urine	Early	Early detection of tubular stress and prediction of progression	Longitudinal studies and meta-analysis	Moderate	Emerging clinical candidate	Limited external validation
**TNFR1/TNFR2**	Inflammation/TNF signaling	Plasma	Early–Progressive	Prediction of kidney function decline, ESKD, and mortality	Large prospective cohorts with independent replication	Strong	Near-clinical implementation	Limited standardization and implementation frameworks
**MCP-1 (CCL2)**	Inflammation	Urine/Plasma	Early	Prediction of microalbuminuria and adverse renal outcomes	Cohort studies and ROADMAP analysis	Limited–Moderate	Emerging clinical candidate	Not kidney-specific; influenced by systemic inflammation
**IL-18**	Inflammasome activation	Urine	Early	Marker of inflammatory kidney injury	Cohort studies and mechanistic evidence	Limited	Experimental	Limited specificity and validation
**suPAR**	Immune activation	Plasma	Early–Progressive	Risk stratification and disease progression assessment	Observational and biopsy-based studies	Limited–Moderate	Emerging clinical candidate	Limited prospective validation
**TGF-β1**	Fibrosis	Plasma/Urine	Progressive	Reflects profibrotic activity and disease progression	Clinical and biopsy studies	Limited	Experimental	Complex biology and lack of standardized thresholds
**CTGF**	Fibrosis	Plasma/Urine	Advanced	Associated with fibrosis burden and ESKD risk	Clinical association studies	Limited	Experimental	Limited utility for early disease detection
**Extracellular vesicles**	Multi-pathway injury	Urine	Very early	Molecular characterization of renal injury	Exploratory studies	Not established	Research-use only	Lack of standardization and reproducibility
**Urinary miRNAs/lncRNAs**	Epigenetic regulation	Urine	Very early	Early molecular signatures of DKD and precision phenotyping	Exploratory studies and meta-analyses	Not established	Research-use only	Technical variability and normalization challenges

**Abbreviations:** CTGF, connective tissue growth factor; DKD, diabetic kidney disease; ESKD, end-stage kidney disease; IgG, immunoglobulin G; IgM, immunoglobulin M; IL-18, interleukin-18; KIM-1, kidney injury molecule-1; L-FABP, liver-type fatty acid-binding protein; MCP-1, monocyte chemoattractant protein-1; miRNA, microRNA; lncRNA, long non-coding RNA; NGAL, neutrophil gelatinase-associated lipocalin; suPAR, soluble urokinase plasminogen activator receptor; TNFR, tumor necrosis factor receptor; UACR, urinary albumin-to-creatinine ratio.

Clinical readiness categories were assigned according to the extent of prospective validation, replication across independent cohorts, assay standardization, and evidence of incremental prognostic value beyond established clinical markers such as urinary albumin-to-creatinine ratio (UACR) and estimated glomerular filtration rate (eGFR).

Box 1 **Key recommendations for clinicians.**

- **UACR and eGFR remain the cornerstone biomarkers** for the diagnosis, staging, and monitoring of diabetic kidney disease and should continue to guide routine clinical decision-making.
- **TNFR1 and TNFR2 currently show the strongest prognostic evidence among emerging biomarkers**, consistently predicting kidney function decline, progression to end-stage kidney disease, and mortality independent of conventional risk factors.
- **NGAL and KIM-1 are the most clinically mature tubular injury biomarkers** and may provide complementary information on tubulointerstitial injury and disease progression.
- **L-FABP, nephrin, transferrin, MCP-1, and suPAR are promising adjunctive biomarkers**, but require further multicenter validation, assay harmonization, and demonstration of incremental clinical utility before routine implementation.
- **IL-18, TGF-β1, and CTGF remain investigational**, with current evidence insufficient to support clinical use outside research settings.
- **Podocyte-derived proteins, extracellular vesicles, and urinary non-coding RNAs should currently be considered research-use biomarkers**, although they may contribute to future precision-nephrology approaches.
- **No emerging biomarker currently has sufficient evidence to replace UACR or eGFR.** The most realistic near-term application is incorporation into multimarker risk-stratification models integrating glomerular, tubular, inflammatory, and fibrotic pathways.
- **Future research should prioritize assay standardization, external validation across diverse populations, demonstration of cost-effectiveness, and evaluation of clinical utility in biomarker-guided therapeutic strategies.**

### 5.6. Non-Albuminuric Diabetic Kidney Disease: Implications for Biomarker-Based Detection and Risk Stratification

The recognition of NADKD has challenged the traditional paradigm that albuminuria represents the earliest clinical manifestation of diabetic renal injury [183,184]. Growing evidence shows that a substantial proportion of patients develop progressive renal dysfunction despite normal urinary albumin excretion, suggesting that tubular injury, inflammation, and impaired reparative mechanisms may precede or occur independently of glomerular permeability changes [36].

Among the biomarkers investigated in NADKD, markers of tubular injury have shown particular promise. NGAL is one of the most extensively studied biomarkers and is frequently elevated in normoalbuminuric diabetic patients [185,186]. Although individual studies have reported high diagnostic accuracy, pooled analyses indicate only moderate sensitivity and specificity, and the absence of standardized cut-off values currently limits widespread clinical implementation [187,188]. Similarly, KIM-1 and L-FABP have been associated with early tubular damage and progressive renal dysfunction, independent of albuminuria [111,183,189,190,191,192,193]. These biomarkers provide complementary information by reflecting different aspects of tubular stress, epithelial injury, and lipid-mediated toxicity, linked to early renal structural damage and subsequent loss of kidney function even in the absence of albuminuria, highlighting their potential for identifying patients at increased risk of disease progression before conventional markers become abnormal. Despite promising evidence, these biomarkers have not yet been incorporated into routine clinical practice and require further validation in large prospective studies.

Cystatin C represents an important filtration marker in NADKD. Unlike creatinine, its concentration is minimally influenced by muscle mass and may detect early reductions in glomerular filtration before overt albuminuria develops. Elevated cystatin C levels have been reported in normoalbuminuric diabetic patients with impaired renal function, supporting its utility for early identification of renal decline and risk stratification in this population [194,195].

Inflammatory and reparative biomarkers further support the concept that NADKD is driven by mechanisms extending beyond glomerular injury. uMCP-1, a key mediator of macrophage recruitment and intrarenal inflammation, has demonstrated strong associations with both renal function decline and disease progression [196]. Likewise, urinary epidermal growth factor (uEGF), a marker of tubular regenerative capacity, and the uEGF/MCP-1 ratio provide insight into the balance between ongoing injury and renal repair [105,197,198,199]. Additional markers, including urinary vitamin D-binding protein (uVDBP) and urinary heat shock protein-72 (uHSP72), have shown encouraging diagnostic performance in normoalbuminuric diabetic patients currently showing the most consistent sensitivity-specificity profiles among individual analytes, implicating disrupted repair-inflammation coupling as a central, and possibly modifiable, driver of nonalbuminuric progression [200,201].

Collectively, these findings suggest that NADKD represents a distinct biological phenotype characterized by predominant tubulointerstitial injury, chronic inflammation, and impaired reparative signaling rather than isolated glomerular damage. While individual biomarkers provide valuable mechanistic information, no single marker fully captures the complexity of the disease. Consequently, composite biomarker approaches, particularly proteomic classifiers such as CKD273, have emerged as promising tools for identifying high-risk normoalbuminuric patients and improving risk prediction beyond albuminuria alone. The growing recognition of NADKD therefore reinforces the need for multimarker strategies that can detect renal injury earlier and enable more precise risk stratification. Table 3 details the clinical readiness of these candidate biomarkers.

**Table 3:** Clinical readiness and translational potential of emerging biomarkers in diabetic kidney disease.

Biomarker	Evidence Level	Independent Validation	Added Value Beyond UACR/eGFR	Commercial Assay Availability	Clinical Feasibility	Main Limitation	Current Readiness
TNFR-1	High	Multiple cohorts	Strong	Yes	High	Limited biomarker-guided intervention data	Closest to implementation
TNFR-2	High	Multiple cohorts	Strong	Yes	High	Limited biomarker-guided intervention data	Closest to implementation
CKD273	High	Multicenter validation	Strong	Limited availability	Moderate	Cost, accessibility, specialized platform	Advanced validation
KIM-1	Moderate–High	Several cohorts	Moderate	Yes	High	Assay standardization and variable effect size	Emerging
NGAL	Moderate	Multiple studies	Moderate	Yes	High	Limited DKD specificity; stage-dependent performance	Emerging
L-FABP	Moderate	Several cohorts	Moderate	Limited/region-dependent	Moderate	Limited external validation and assay availability	Emerging
suPAR	Moderate	Growing evidence	Moderate	Yes	Moderate	Uncertain DKD specificity; influenced by systemic inflammation	
Nephrin	Low–Moderate	Limited replication	Potentially high	Limited	Moderate	Limited replication and assay standardization	Investigational
EV-derived biomarkers	Moderate	Limited	Potentially high	No	Low–Moderate	Lack of standardized isolation and analysis methods	Emerging research
miRNA panels	Early–Moderate	Limited	Potentially high	No	Low	Reproducibility, normalization, and platform variability	Research stage
Metabolomic signatures	Moderate	Several cohorts/meta-analyses	Potentially high	No	Low–Moderate	High analytical complexity and limited DKD specificity	Research stage

Evidence levels are qualitative and based on consistency of findings, availability of prospective studies, replication in independent cohorts, incremental value beyond UACR/eGFR, assay availability, feasibility, and current translational maturity. UACR, urinary albumin-to-creatinine ratio; eGFR, estimated glomerular filtration rate; TNFR, tumor necrosis factor receptor; CKD273, urinary proteomic classifier; KIM-1, kidney injury molecule-1; NGAL, neutrophil gelatinase-associated lipocalin; L-FABP, liver-type fatty acid-binding protein.

Although individual biomarkers and targeted biomarker panels provide valuable insights into specific pathogenic pathways, they capture only a limited subset of the complex molecular landscape of DKD. This constraint has led to growing interest in multi-omics approaches, which integrate data from multiple biological layers to facilitate a more comprehensive characterization of disease processes.

## 6. From Single Biomarkers to Multi-Omics Signatures in DKD

In addition to studying single biomarkers, there has been a growing trend in recent DKD research towards multi-omics approaches. This direction is sensible, since DKD is not a disease that can be explained by one pathway or one structural compartment of the kidney. It includes glomerular, tubular, vascular, inflammatory, fibrotic, mitochondrial and metabolic changes that may occur at different times and with different severity among patients. Therefore, the complexity of this process cannot be represented by a single urinary or circulating marker.

Liquid biopsy-based multi-omics technologies enable non-invasive assessment of multiple biological layers, including RNA expression, urinary proteins and peptides, extracellular vesicle cargo, lipid remodeling and metabolite signatures [202,203,204]. Urine is of particular interest in this context, because it may contain molecular signals directly derived from tubular cells, podocytes, endothelial cells and inflammatory cells involved in renal injury. Thus, omics approaches based on urine may offer a more direct insight into intrarenal processes than many systemic biomarkers.

Integration of transcriptomic, proteomic, extracellular vesicle and metabolomic data may provide a more integrated view of disease activity than albuminuria and estimated glomerular filtration rate (eGFR) alone [205,206]. However, it is worth noting that this field is still largely in the biomarker discovery and validation phase. Most of these markers are not ready for clinical decision-making for routine purposes. The main limitations are related to differences in sample collection and storage, analytical platforms, normalization strategies, bioinformatic pipelines, costs and limited external validation in different populations [204,206]. Thus, multi-omics approaches at present should be viewed as complementary tools for improved biological phenotyping and risk stratification, not as replacements for albuminuria and eGFR.

### 6.1. MicroRNAs (miRNAs)

MicroRNAs (miRNAs) are a class of small non-coding RNAs that function at the post-transcriptional level to control gene expression. They are of particular interest in DKD as they may represent early regulatory changes involved in fibrosis, inflammation, oxidative stress, endothelial dysfunction, podocyte injury and tubular stress [12,207]. Unlike albuminuria and eGFR, which are typically markers of established functional or structural damage, miRNA changes could occur earlier in the course of disease. This makes them attractive as potential early biomarkers, but also as markers of active molecular pathways.

Several miRNAs have been linked to DKD, including miR-21, miR-29, miR-192, and miR-377 [207]. However, these candidates should not be interpreted as having the same level of clinical validation. One of the most studied miRNAs is miR-21, which has a strong mechanistic link with inflammation and renal fibrosis. It is involved in TGF-β signaling, extracellular matrix accumulation and fibrotic remodeling pathways. However, miR-21 is not specific for DKD as it is also altered in other types of CKD and other fibrotic conditions [207]. Thus, miR-21 is likely a marker of active injury and fibrosis and not a specific diagnostic marker of DKD.

The miR-29 family, especially miR-29c, is more related to regulation of extracellular matrix and early fibrotic changes. The role of miR-29 is biologically meaningful, as fibrosis is one of the final common pathways of progressive kidney damage. miR-192 has been consistently associated with diabetic renal injury and its interpretation seems more informative when considered in conjunction with miR-29c. In contrast, miR-377 is also mechanistically interesting, notably in relation to oxidative stress, mesangial expansion and matrix accumulation, but has a lower level of clinical validation than miR-21, miR-29 and miR-192 [207].

Taken together, these four candidates occupy distinct positions along the validation pathway rather than representing a single, interchangeable class of biomarker. miR-21 is the most mechanistically characterized, with the largest body of supporting data, but its lack of DKD specificity confines it to the role of a general fibrosis/injury marker rather than a diagnostic candidate. miR-192, particularly in combination with miR-29c, currently shows the most promising diagnostic performance and the closest approach to a validated, combination-based candidate, whereas miR-29c alone and miR-377 remain at an earlier, exploratory stage of validation, supported chiefly by mechanistic plausibility rather than independently replicated diagnostic accuracy data. Ranked by stage of clinical validation, the evidence therefore places the miR-192/miR-29c combination furthest along, miR-21 as mechanistically important but non-specific, and miR-29c alone and miR-377 as the least validated of the four [207].

A recent systematic review and meta-analysis showed that miRNAs had a pooled sensitivity of 0.76, specificity of 0.74 and area under the receiver operating characteristic curve (AUC) of 0.79 for early detection of DKD [208]. Importantly, urinary miRNAs were more responsive than blood derived miRNAs supporting the concept that urine may be more representational of molecular damage in the kidney [208]. The combined miR-192 and miR-29c analysis demonstrated a better diagnostic performance with a reported sensitivity and specificity of 0.92 and 0.89, respectively [209]. However, this finding should be interpreted with caution because it is based on a small number of research and requires additional validation.

Thus, miRNAs are not yet sufficient to be used as biomarkers in ordinary clinical practice. They are more accurately described as biomarkers for research usage that may be part of future multimarker panels. However, there are still some technical challenges not overcome such as the varied sample sources, different RNA extraction methods, different quantitative PCR processes, lack of uniform endogenous controls and uneven normalization methodologies [12,208]. Moreover, many miRNAs are not kidney-cell-specific and may reflect systemic inflammation, vascular damage or diabetes-related metabolic stress rather than DKD per se. Therefore, prospective studies are needed to assess the top miRNA candidates, to validate them and to test whether they provide any genuine predictive value over albuminuria, eGFR and proven tubular or inflammatory indicators.

### 6.2. Proteomic Signatures in Diabetic Kidney Disease: Diagnostic, Prognostic, and Therapeutic Implications

Proteomics has arisen as a powerful approach for clarifying the molecular complexity of DKD by enabling large-scale characterization of proteins, their post-translational modifications, and dynamic interactions within biological systems [210,211]. Among proteomic approaches, urinary proteomics has attracted particular interest because urine represents a non-invasive “liquid biopsy” that directly reflects ongoing intrarenal molecular and structural alterations. Using high-resolution techniques such as capillary electrophoresis coupled with mass spectrometry (CE-MS), several urinary peptide and protein signatures associated with inflammation, fibrosis, extracellular matrix remodeling, and tubular injury have been identified in DKD [175].

One of the most extensively validated urinary proteomic classifiers is CKD273, a panel composed of 273 urinary peptides initially identified in 2010 [210].

Comprising 273 urinary peptides that predominantly reflect extracellular matrix remodeling, collagen turnover, inflammation, and tubular injury, CKD273 was developed to identify molecular alterations associated with renal damage before the emergence of conventional clinical markers such as albuminuria or a decline in eGFR. The development of this classifier emerged from the recognition that DKD is characterized by complex molecular changes that precede overt clinical manifestations by several years. Early investigations demonstrated that urinary peptide signatures could identify diabetic individuals at increased risk of progressive renal dysfunction and worsening albuminuria before clinically detectable disease became apparent [212,213]. Subsequent studies linked these proteomic alterations to future eGFR decline, chronic kidney disease progression, and histologically confirmed renal fibrosis, providing biological evidence that urinary proteomics reflects underlying pathological processes rather than merely established renal injury [214,215].

Multicenter validation studies have further established the clinical relevance of CKD273 by demonstrating reproducible performance across various laboratories and analytical platforms [216]. This analytical robustness is a critical step toward clinical translation, addressing the frequent limitation in biomarker discovery where promising results lack reproducibility outside initial development cohorts. As a result, CKD273 now serves as the benchmark for evaluating newer proteomic biomarkers.

CKD273 has significantly challenged the traditional albuminuria-centered model of DKD. While albuminuria has long been regarded as the earliest clinically detectable sign of diabetic kidney injury, multiple studies have shown that elevated CKD273 scores identify individuals at increased risk of disease progression even when urinary albumin excretion remains within normal limits [217,218].

In 1014 normoalbuminuric individuals with preserved renal function, CKD273 independently predicted progression to eGFR < 60 mL/min/1.73 m^2^, and this association was not explained by age, blood pressure, or baseline eGFR [212]; in a separate prospective cohort of 1775 normoalbuminuric patients with type 2 diabetes, a high-risk CKD273 profile predicted incident microalbuminuria over a median 2.5-year follow-up, independent of glycemic control and blood pressure [218].

The analytic significance of these findings is that CKD273 outperforms albuminuria specifically in the population where albuminuria is, by definition, uninformative: normoalbuminuric patients. This is a qualitatively different claim from showing that a marker correlates with albuminuria in albuminuric cohorts, and it is the reason proteomic classifiers are now positioned as the most clinically advanced tool for NADKD risk stratification, a status reinforced by the U.S. Food and Drug Administration’s encouragement of further CKD273 qualification studies [212,218].

The prognostic value of CKD273 has been most clearly demonstrated in prospective outcome studies. For example, the multinational PRIORITY study, which included 1775 normoalbuminuric individuals with type 2 diabetes, showed that CKD273 could identify a subgroup at significantly increased risk of developing microalbuminuria and experiencing renal function decline, despite normal baseline urinary albumin excretion [218]. These results provide prospective evidence that urinary proteomic profiling can detect ongoing kidney injury during a clinically silent phase. The study also highlighted the distinction between prognostic enrichment and therapeutic efficacy. Although spironolactone did not significantly reduce progression to microalbuminuria among CKD273-defined high-risk individuals, the classifier effectively identified a population enriched for future renal events [218]. This capacity to stratify risk before clinical deterioration is a key application of proteomics in precision medicine.

CKD273 also demonstrates prognostic relevance for cardiovascular disease and mortality. Multiple studies have shown that urinary proteomic profiling enhances prediction of cardiovascular events and all-cause mortality beyond conventional clinical variables in patients with diabetes and early chronic kidney disease [219,220]. These findings suggest that the molecular pathways identified by CKD273 extend beyond the kidney and reflect broader mechanisms underlying systemic vascular injury and cardiorenal disease progression. This evidence supports the concept that diabetic kidney disease and cardiovascular disease share common pathogenic pathways, including inflammation, fibrosis, endothelial dysfunction, and extracellular matrix remodeling.

CKD273 has emerged as a promising and potentially cost-effective biomarker for the early detection and risk stratification of diabetic kidney disease (DKD). Compared with urinary albumin excretion testing, it demonstrated favorable cost-effectiveness, with an ICER of approximately EUR 23,900 per QALY gained, particularly among high-risk patients [221]. By identifying renal injury before overt clinical manifestations, CKD273 may facilitate earlier intervention, delay progression to end-stage kidney disease, reduce dialysis-related costs, and improve quality of life. Findings from the PRIORITY studies further support its utility in guiding targeted preventive therapies [218,222].

However, its predictive performance appears strongest in the early stages of DKD, while its ability to predict progression to advanced CKD or macroalbuminuria has been less consistent [223]. Although CKD273 correlates with established markers such as eGFR and albuminuria, evidence that CKD273-guided management improves long-term renal outcomes remains limited. In addition, implementation requires specialized capillary electrophoresis–mass spectrometry technology that is not widely available in routine clinical practice. Furthermore, most economic evaluations have been conducted in European healthcare settings, which may limit the generalizability of findings. Therefore, further large-scale interventional and health–economic studies are needed before widespread clinical adoption can be recommended.

Proteomics is increasingly being investigated for its potential role in therapeutic monitoring. Post hoc analyses from the MARLINA-T2D trial indicated that linagliptin treatment may induce changes in urinary peptide profiles, potentially identifying individuals more likely to benefit from therapy [224]. Similarly, urinary proteomic analyses after empagliflozin administration revealed alterations in peptides related to collagen metabolism, clusterin, and mucin-related pathways, resulting in a proteomic profile resembling that of individuals with preserved renal health [225]. Notably, CKD273 scores were not significantly altered during short-term treatment, suggesting that while the classifier captures overall disease risk, it may not fully reflect all biological pathways targeted by specific therapies [225]. Comparable results have been observed with GLP-1 receptor agonists, which induced significant changes in urinary peptides associated with collagen turnover, inflammation, and insulin resistance pathways [226]. Collectively, these findings indicate that proteomics may contribute to both risk stratification and the selection and monitoring of therapeutic responses.

While CKD273 remains the most mature proteomic biomarker in DKD, numerous emerging biomarkers have enhanced understanding of disease pathophysiology and may offer complementary prognostic information. Proteomic studies increasingly highlight extracellular matrix remodeling, inflammation, complement activation, and tubular injury as central processes in disease progression. Integrative analyses that combine urinary proteomics, kidney tissue proteomics, and transcriptomics have consistently identified changes in collagens, annexins, cathepsins, thrombospondin-1, and galectin-3, underscoring the central role of dysregulated extracellular matrix turnover and fibrosis in DKD pathogenesis [227]. The consistency of these findings across multiple platforms supports the view that urinary proteomic signatures reflect fundamental disease mechanisms rather than secondary effects of established kidney damage.

Complement activation is a particularly notable emerging pathway. Large translational studies across multiple cohorts have shown strong associations between urinary complement proteins and subsequent renal disease progression, risk of kidney failure, and histopathological severity [228,229]. These associations are stronger in urine than in plasma, suggesting that urinary complement proteins more accurately reflect intrarenal pathological processes. Increased complement protein abundance and complement-related gene expression in diabetic kidney tissue further support the role of complement activation as a biologically relevant contributor to disease progression, rather than merely a biomarker of injury [228].

Inflammation is another major focus of proteomic discovery. Chronic low-grade inflammation is increasingly recognized as a key driver of fibrosis, endothelial dysfunction, oxidative stress, and progressive kidney function loss in DKD [230]. The Kidney Risk Inflammatory Signature (KRIS), which includes 17 circulating inflammatory proteins, has shown strong associations with renal function decline and progression to end-stage renal disease (ESRD) in both type 1 and type 2 diabetes [231]. These associations are largely independent of albuminuria, indicating that inflammatory pathways provide prognostic information not captured by traditional biomarkers. These findings support the view that DKD comprises multiple biological endotypes that may not be adequately characterized by albuminuria alone.

Additional candidate biomarkers are emerging from proteomic discovery studies. Uromodulin, a kidney-specific protein indicative of tubular function, has been shown to increase before the onset of microalbuminuria and may serve as an early marker of tubular dysfunction in diabetes [232]. Similarly, urinary cathepsin D is associated with rapid eGFR decline and more severe tubulointerstitial inflammation, suggesting its potential as both a biomarker of disease activity and a marker of maladaptive cellular responses to the diabetic microenvironment [233]. Although these biomarkers require further validation, they offer important mechanistic insights into the heterogeneity of DKD pathogenesis.

Advances in machine learning and large-scale proteomics have accelerated biomarker discovery. Machine-learning algorithms applied to urinary proteomic datasets have identified multimarker panels that include proteins involved in inflammatory, metabolic, complement-mediated, and fibrotic pathways, achieving strong diagnostic performance in validation cohorts [234]. Similarly, plasma proteomic studies using high-throughput platforms such as SOMAscan have identified circulating proteins, including LAYN, ESAM, DLL1, MAPK11, and endostatin, as independent predictors of progression to kidney failure [235]. Incorporating these proteomic markers into conventional clinical models significantly improves risk prediction, indicating that circulating and urinary proteomics may provide complementary information on disease susceptibility and progression.

Researchers are increasingly integrating proteomic, genomic, transcriptomic, metabolomic, and clinical data to develop multidimensional prediction tools that identify disease susceptibility and progression earlier and more accurately than conventional markers alone [236,237]. These approaches are closely aligned with the principles of precision nephrology, which incorporate biological heterogeneity into risk assessment and therapeutic decision-making.

Despite these advances, most emerging proteomic biomarkers remain in early stages of clinical development, and none have achieved the level of validation or translational maturity demonstrated by CKD273 [216,217,218,220,221].

Collectively, these developments reflect a research trend in DKD biomarker discovery away from the identification of individual markers toward integrated molecular profiling approaches that capture the complexity and heterogeneity of disease progression. While CKD273 remains the most extensively validated proteomic classifier and serves as the benchmark for urinary proteomics, emerging biomarkers related to complement activation, inflammatory signaling, extracellular matrix remodeling, plasma proteomics, and machine-learning-derived signatures continue to expand understanding of DKD pathophysiology and risk stratification. However, a significant gap persists between biomarker discovery and clinical implementation. Most proteomic approaches have improved risk prediction but have not yet demonstrated definitive improvements in patient outcomes. Therefore, future research should focus on refining molecular classifiers and establishing their clinical utility through prospective interventional studies to determine whether biomarker-guided management can alter disease trajectories. Whether these approaches ultimately contribute to precision nephrology will depend on whether molecular insights can be translated into validated, prospectively tested clinical strategies, although, at present, this remains an open question rather than an established outcome.

### 6.3. Metabolomic Profiling in Diabetic Kidney Disease: From Molecular Mechanisms to Precision Medicine

Metabolomics is a branch of omics science focused on the comprehensive analysis of low-molecular-weight compounds (<1500 Da) involved in cellular metabolism within biological fluids and tissues [238]. By identifying the origin, dynamics, and concentration changes of these metabolites, metabolomics offers valuable insights into the physiological state and functional activity of biological systems. As metabolites are the end products of gene expression and protein activity, metabolomics provides a functional readout that closely reflects phenotype and integrates both genetic and environmental factors [239,240,241,242]. Urine metabolomics is particularly attractive in DKD because it enables non-invasive assessment of renal metabolic alterations and may directly reflect pathological changes occurring within the kidney.

Metabolomic analyses consistently reveal disruptions in key metabolic pathways in DKD, including the tricarboxylic acid (TCA) cycle, amino acid metabolism, lipid metabolism, and nucleotide metabolism [243]. These metabolic alterations are strongly associated with mitochondrial dysfunction, now recognized as a central factor in DKD pathogenesis [244]. Disrupted mitochondrial energy metabolism, along with oxidative stress and inflammation, appears to drive progressive renal injury [243,245].

Several studies also demonstrate the utility of metabolomics in distinguishing DKD from other renal disorders in diabetic patients, underscoring its potential to improve disease characterization, diagnosis, and prognosis [242].

A key question for diagnostic utility is whether these metabolite signatures are specific to DKD or instead reflect shared metabolic disturbances common to chronic kidney disease (CKD) of any etiology. Mitochondrial dysfunction, TCA cycle disruption, and amino acid metabolism changes, the most consistently reported alterations, are not unique to diabetes and have also been described in non-diabetic CKD, where reduced GFR and tubular dysfunction independently affect systemic metabolite clearance and handling. This overlap means that several of the most frequently cited individual metabolites are better understood as markers of reduced kidney function in general rather than markers specific to the diabetic pathophysiological process. Stronger evidence for genuine DKD specificity comes from studies that directly compare diabetic and non-diabetic kidney disease cohorts rather than diabetic patients against healthy controls; this includes the tryptophan–kynurenine pathway and select purine metabolites, which have been reported to differentiate DKD from other renal conditions when such comparator groups are used. By contrast, many of the broader pathway-level signals identified in case-control studies against healthy individuals cannot currently be assumed to be DKD-specific, since these designs cannot distinguish a diabetes-driven mechanism from a generic consequence of impaired renal clearance. For diagnostic utility, this distinction matters: metabolites that merely track eGFR have limited value as DKD-specific biomarkers, whereas a smaller subset that differentiates DKD from other CKD etiologies in head-to-head comparisons would have genuine discriminative value, for example in distinguishing diabetic from non-diabetic kidney disease in patients with diabetes and atypical clinical presentations. Future studies should therefore prioritize non-diabetic CKD comparator groups when evaluating candidate metabolite panels, rather than relying solely on comparisons with metabolically healthy controls.

One of the first metabolomic investigations evaluating eGFR decline in patients with T2DM was conducted by Ng et al., who analyzed the metabolome of 90 non-proteinuric patients and identified the best-performing metabolite subsets using LASSO logistic regression. Their results showed that 11 metabolites were significantly associated with eGFR decline. In particular, oxalic acid was detected in 95.6% of the control subjects but was absent in patients with reduced eGFR, consistent with the systemic retention of this metabolite in individuals with impaired renal function [246].

Emerging data have identified metabolite signatures, particularly in the urea cycle, tryptophan–kynurenine pathway, purine metabolism, and lipid metabolism, that differentiate DKD from other renal conditions [247,248,249]. Recent studies with biopsy-confirmed DKD show that integrating metabolomic profiles with clinical parameters significantly improves diagnostic accuracy, underscoring the clinical relevance of these approaches [250]. A large longitudinal analysis from the DPPOS cohort extended metabolomic risk assessment to individuals with prediabetes, demonstrating that metabolic profiles associated with DKD differ from those linked to other microvascular complications. Certain biomarkers predicted nephropathy risk only under specific therapeutic conditions, highlighting the potential of metabolomics for early risk stratification and personalized intervention before the onset of overt diabetes [251]. These findings indicate that metabolomics can provide disease-specific molecular signatures beyond conventional clinical markers [239].

Beyond diagnosis, metabolomics shows significant potential for predicting disease progression. Large-scale and longitudinal studies have identified metabolite panels linked to rapid kidney function decline, independent of traditional risk factors [116,252]. Specific metabolites, including 3-hydroxyisobutyrate, aconitic acid, and myo-inositol, are associated with changes in eGFR trajectory and risk of progression to ESKD [252,253]. These biomarkers often provide additional predictive value when combined with established clinical indicators such as albuminuria and baseline renal function [253]. Advanced analytical approaches, including machine-learning methods, have further improved identification of metabolite-based predictors of disease progression [254].

A recent meta-analysis of 39 longitudinal studies (over 31,000 participants) identified 76 metabolites significantly associated with DKD onset and progression. These biomarkers are mainly involved in amino acid, lipid, and energy metabolism, highlighting substantial metabolic reprogramming in DKD [255].

Emerging evidence suggests that DKD shares metabolic features with other diabetic microvascular complications, especially diabetic retinopathy (DR). Alterations in pyrimidine metabolism, amino acid metabolism, and the pentose phosphate pathway have been observed in both conditions [256,257,258]. Certain metabolites, such as N-acetylneuraminic acid, are associated with both renal and retinal complications, indicating common mechanisms and reinforcing the systemic nature of diabetic microvascular disease [259,260].

Integrating metabolomics with other omics platforms, such as genomics, transcriptomics, and proteomics, is critical for a comprehensive understanding of DKD. Multi-omics approaches identify complex molecular networks and interactions that single-layer analyses cannot capture [258]. Table 4 presents an overview of current multi-omics biomarkers in diabetic kidney disease, including their underlying molecular mechanisms, level of evidence, and key translational challenges.

Recent studies using multiple omics datasets have highlighted key pathways, including lipid metabolism dysregulation, and improved identification of individuals at high risk of DKD through network-based and machine-learning approaches [261,262]. These integrative strategies may eventually inform precision medicine and targeted therapy development, but remain at an early, hypothesis-generating stage and require substantial further validation.

Several challenges limit the clinical translation of metabolomics. Technical issues include incomplete metabolome coverage, variability in analytical platforms, and lack of standardized protocols for sample processing and data analysis [263,264]. Biological variability from genetic, environmental, and lifestyle factors further complicates data interpretation. No single metabolite has been identified as a universally reliable biomarker for DKD, highlighting the disease’s complexity and heterogeneity [264].

Taken together, metabolomics provides useful mechanistic insights into the biochemical alterations of underlying DKD. However, its candidate diagnostic and prognostic applications, including any contribution to precision-medicine approaches when integrated with multi-omics data, remain investigational. Large-scale validation studies and methodological standardization are required before routine clinical implementation [204,265]. Table 4 compares these approaches by molecular mechanism and clinical readiness.

**Table 4:** Multi-omics biomarkers of diabetic kidney disease: molecular mechanism, level of evidence and translational challenges.

Biomarker/Omics Platform	Representative Biomarkers/Signatures	Primary Biological Processes Represented	Reported Performance and Evidence	Current Interpretation in DKD	Major Constraints and Translational Challenges
Transcriptomics/RNA analysis	lncRNAs, miR-21, miR-29, miR-192, miR-377	Control of fibrosis, inflammation, oxidative stress, podocyte injury, endothelial dysfunction and extracellular matrix remodeling.	A recent meta-analysis showed that the sensitivity and specificity of miRNAs for early DKD were 0.76 and 0.74, respectively, and the AUC was 0.79 [208]. Combined miR-192 + miR-29c had sensitivity and specificity of 0.92 and 0.89, respectively, but with limited data [208].	Mostly used as research-use biomarkers. miR-21 is mechanistically powerful but not DKD-specific. miR-29c and miR-192 seem more promising in combination.	Small and varied studies, multiple sample sources, different RNA extraction and normalization procedures, no consensus cut-offs, low cell-type specificity and uncertain DKD specificity.
Proteomics	CKD273 urinary peptide classifier	Remodeling of the extracellular matrix, fibrosis, tubular damage, inflammation and vascular dysfunction.	The most validated urinary proteomics classifier for DKD. In the PRIORITY trial, 1775 type 2 diabetes patients were enrolled to identify patients at increased risk before the onset of microalbuminuria using CKD273 [218,266].	It is the most clinically advanced omics-based classifier to date, but should be used as an addition to albuminuria, eGFR and clinical risk factors rather than a stand-alone test.	High cost, limited availability of CE-MS, requirement for harmonized processes, variable performance in clinical settings and currently undetermined direct impact on treatment decisions.
Proteomics	KRIS inflammatory signature	Chronic inflammation, immunological activation, endothelial dysfunction and gradual renal deterioration.	A total of 17 circulating inflammatory proteins were linked to progressive renal deterioration and ESRD in type 1 and type 2 diabetes [231].	An important feature of prognostic research. It supports the role of inflammation in DKD progression but is not considered DKD-specific.	Inflammatory proteins may also reflect systemic inflammation, cardiovascular disease, infection or concomitant CKD. Further external confirmation is required.
Proteomics	Collagen peptides in urine	Tissue remodeling and renal fibrosis; extracellular matrix turnover.	Urinary collagen-derived peptides have been linked to biopsy-proven renal fibrosis [215].	Potential non-invasive measure of structural damage and fibrosis burden. In clinical practice, it is typically employed as part of a wider proteomic profile.	It requires specialized platforms, careful bioinformatic interpretation and subsequent validation using histology and long-term renal outcomes.
Extracellular vesicles/tubular indicators/proteomics	Urinary exosomal UMOD mRNA and uromodulin	Tubular integrity, tubular stress and early tubular dysfunction.	In 100 participants, Barr et al. reported increased urinary exosomal UMOD mRNA and urinary uromodulin in patients with type 2 diabetes prior to microalbuminuria [232].	A potential indication of early tubular involvement in kidney disease. It should be described as a research biomarker at this time, not a proven clinical diagnostic.	Small sample size, unclear cut-offs, possible differences among CKD stages and need for prospective validation in other populations.
Extracellular vesicles	EV podocyte and tubular cell cargo: mRNA, miRNA, proteins and lipids	Cell-to-cell communication, podocyte injury, tubular stress, inflammation, fibrosis, oxidative stress and cellular healing mechanisms.	Urinary EVs are increasingly explored for liquid biopsy in DKD. The data are robust in a biological sense but largely exploratory [209,232]	Can integrate transcriptomic and proteomic data from kidney cells. Could be valuable for early phenotyping and mechanistic research.	Strong pre-analytical sensitivity: urine collection, hydration, time of day, activity, nutrition, glycosuria, UTI, hematuria, storage, isolation method, RNA extraction and normalization may affect the results.
Proteomics	Complement proteins (CFH, C2, C5a, C6, C7)	Activation of complement, inflammation, endothelial injury and immune-mediated kidney damage.	Urinary proteomic data prospectively associated complement proteins with DKD progression, with validation in an independent cohort [228].	May contribute to the improvement of future prognostic models, especially in patients with inflammatory or complement-related patterns of progression.	Assay standardization, clinical thresholds, disease specificity and integration into routine decision-making are still to be determined.
Proteomics	Cathepsin D	Lysosomal dysfunction, tubular stress and tubulointerstitial inflammation.	In type 1 diabetes, urinary cathepsin D peptides and protein levels were correlated with rapid reduction in eGFR and more severe tubulointerstitial inflammation [233].	Potential indication of a more aggressive tubulointerstitial phenotype of DKD.	Limited large-scale validation, limited specificity and questionable added value over established tubular markers.
Metabolomics	TCA metabolites/mitochondrial energy metabolites	Mitochondrial malfunction, energy metabolism alterations, oxidative stress and metabolic reprogramming.	Repeatedly reported in DKD metabolomic investigations, but most signals suggest pathway-level metabolic stress, not a particular DKD-specific molecule [243,244,245].	Useful for understanding renal metabolic stress and for creating risk panels, particularly when paired with clinical factors.	High biological variability and impact of food, medication, glucose control, renal function and platform differences.
Metabolomics	Tryptophan metabolites in the kynurenine pathway	Inflammation, immunological activation, endothelial dysfunction and oxidative stress.	Reported in investigations of DKD and CKD development as part of inflammatory and metabolic markers [247,248,249].	Potential pathway marker, more valuable for mechanistic interpretation than DKD-specific diagnosis.	Not DKD-specific. May be influenced by systemic inflammation, microbiota activity, reduced renal clearance and comorbidity.
Metabolomics	Myo-inositol	Tubular malfunction, osmotic stress and defective glucose-linked metabolism.	Associated with trajectories of eGFR and risk of progression to ESRD in long-term studies [252,253].	Best viewed as part of a panel of metabolites, not in isolation. Candidate prognostic metabolite.	Must be confirmed in larger and ethnically diverse cohorts. Thresholds and standardization of assays are not well established.
Metabolomics	3-hydroxyisobutyrate, aconitic acid and amino acid metabolites	Amino acid metabolism dysregulation, mitochondrial stress, energy metabolism and systemic metabolic damage.	Related to longitudinal deterioration in renal function and likelihood of progression in cohorts [252,253].	May add prognostic risk stratification to UACR, eGFR and clinical factors.	These metabolites are not specific for DKD and repeatability across platforms and cohorts remains to be established.
Metabolomics/lipidomics	Lipid mediators and lipidomic signatures	Lipotoxicity, mitochondrial damage, inflammation, insulin resistance and endothelial dysfunction.	Integrated omics and machine-learning studies have demonstrated dysregulated lipid metabolism in patients at higher risk for DKD [261,262].	May help define high-risk metabolic phenotypes and guide future tailored treatment methods.	Difficult interpretation, cost, limited availability, influence of diet and medicines, and need for external validation.
Integrated multi-omics strategies	Combined transcriptomic, proteomic, EV-derived, metabolomic, lipidomic, genomic and clinical markers	Inflammation, fibrosis, oxidative stress, endothelial dysfunction, mitochondrial injury, tubular stress and extracellular matrix remodeling.	Multi-omics models can improve molecular phenotyping and risk classification in specific cohorts, but performance depends on data quality, cohort composition and validation technique [258,261,262].	Currently exploratory; best used for research, cohort enrichment, and hypothesis generation. May contribute to precision-medicine and systems-nephrology frameworks if validated, but is not yet established for this purpose.	High expense, bioinformatic complexity, risk of overfitting, lack of routine clinical applicability and lack of large-scale validation in non-European and varied populations.

**Abbreviations:** miRNA, microRNA; lncRNA, long non-coding RNA; EV, extracellular vesicle; CKD273, chronic kidney disease 273-peptide classifier; KRIS, Kidney Risk Inflammatory Signature; UMOD, uromodulin; CFH, complement factor H; TCA, tricarboxylic acid; CE-MS, capillary electrophoresis-mass spectrometry; UACR, urinary albumin-to-creatinine ratio; eGFR, estimated glomerular filtration rate; ESRD/ESKD, end-stage renal/kidney disease; DKD, diabetic kidney disease; CKD, chronic kidney disease; UTI, urinary tract infection; AUC, area under receiver operating characteristic curve.

## 7. Clinical Implications

The clinical implications of emerging molecular and multi-omics biomarkers represent a major focus of current research in DKD, particularly for early detection, risk stratification, and therapeutic monitoring. Unfortunately, the majority of these candidates remain investigational, insufficiently standardized, and not yet ready for routine clinical implementation; their principal current value lies in the potential, if validated, to detect disease at a subclinical stage, well before conventional markers become abnormal. Currently, serum creatinine, eGFR and microalbuminuria remain the gold standard for DKD diagnosis and monitoring [39]. However, microalbuminuria reflects an already established degree of glomerular damage and fails to capture the early molecular events underlying disease progression. Urinary biomarkers such as KIM-1 [267], NGAL [268], and microRNAs (miRNAs) [268] have demonstrated utility even during the subclinical phase of the disease.

Beyond early detection, novel biomarkers may also provide prognostic information useful for risk stratification and therapeutic decision-making, areas in which conventional markers remain limited. Traditional biomarkers cannot reliably identify patients at the highest risk of rapid disease progression. Therefore, incorporating circulating and urinary biomarkers into clinical risk scores could represent an important step toward more personalized management of high-risk patients.

In this regard, Schrauben et al. analyzed data from a large cohort of 898 patients and found that higher urinary levels of KIM-1 and MCP-1, biomarkers of tubular injury and inflammation, were associated with a greater risk of CKD progression. In contrast, higher levels of Epidermal Growth Factor (EGF) were associated with a lower risk of adverse renal outcomes [269].

Therefore, incorporating emerging biomarkers into clinical risk models may represent an important step toward more personalized management of high-risk patients. Traditionally, clinicians rely on changes in eGFR slope and albuminuria to evaluate treatment response. However, these parameters change relatively slowly compared with urinary biomarkers, which may provide more sensitive and earlier indications of therapeutic effects. In individualized patient care, serial biomarker measurements could allow clinicians to dynamically assess treatment response and optimize therapeutic strategies more effectively.

This concept was demonstrated by Malijan et al. in their study evaluating the effects of empagliflozin on urinary biomarkers. Among 2700 participants in the EMPA-KIDNEY trial, empagliflozin treatment resulted in a substantial and sustained reduction in UMOD levels, a biomarker associated with eGFR decline, along with moderate increases in urinary α1-microglobulin (A1M) and Dickkopf-related protein 3 (DKK-3) [270,271].

Therefore, integrating these novel biomarkers with traditional diagnostic approaches may significantly improve the early detection of DKD, enabling timely nephroprotective interventions at stages when treatment is most likely to be effective. Moreover, the development of multiplex molecular biomarker panels capable of simultaneously assessing multiple biomarkers from a single urine sample could facilitate frequent, non-invasive monitoring and promote a more proactive, personalized, and precision-based approach to disease management.

## 8. Limitations and Challenges

Despite recent advances and the development of innovative technologies, the diagnosis and management of DKD still face several important challenges before urinary biomarkers can achieve full clinical applicability.

One major limitation is the lack of standardized methodologies and validated clinical thresholds, particularly in proteomics and metabolomics. Novel biomarker approaches are not inherently superior simply because they rely on advanced technologies. Variability in sample collection, processing, storage, and analytical methods across laboratories can substantially affect biomarker measurements, leading to inconsistent and difficult-to-reproduce results [272]. Moreover, the pre-analytical phase alone can significantly influence biomarker concentrations and represents a major source of variability when standardized procedures are lacking [273]. Consequently, until harmonized protocols for sample handling and biomarker analysis are established, comparisons across studies and the translation of these biomarkers into clinical practice will remain challenging.

Another significant limitation is the restricted generalizability of current evidence. Most emerging urinary biomarkers have been assessed in relatively small, single-center, and ethnically homogeneous cohorts, leaving critical questions regarding sex, ethnicity, and disease-stage-specific performance unresolved [274,275]. This issue is particularly relevant in DKD, a highly heterogeneous condition influenced by genetic background, ethnicity, comorbidities, environmental exposures, and variations in clinical management. These factors may independently affect biomarker expression, irrespective of renal injury [276]. Implementation also encounters additional challenges. Many biomarkers lack assay standardization, consistent sample collection and analytical protocols, universally accepted reference ranges, and adequate external validation [277,278,279]. Measured concentrations are susceptible to biological and pre-analytical variables, such as hydration status, dietary intake, physical activity, medication use, and sample handling. Furthermore, the extent to which biomarker changes indicate disease activity, therapeutic response, or both, remains insufficiently understood [280,281]. Addressing these limitations will require large, multicenter, prospective studies in diverse populations to validate biomarker performance across patient groups and to establish clinical value beyond conventional diagnostic markers.

Cost and technological accessibility also represent major barriers to implementation. Healthcare resources are unevenly distributed worldwide, and access to advanced diagnostic technologies remains limited, particularly in resource-constrained settings [282]. Furthermore, validating novel biomarkers can be lengthy and complex. The clinical utility of any emerging diagnostic tool depends not only on its accuracy but also on its scalability, reproducibility, and accessibility. For example, proteomic and metabolomic analyses often require highly specialized laboratory environments, limiting their widespread clinical adoption. Mass spectrometry, despite its ability to simultaneously detect a broad spectrum of proteins and metabolites, remains expensive and technically demanding, making routine implementation difficult [283].

Nevertheless, the history of medical innovation demonstrates that healthcare systems can successfully integrate initially expensive technologies when their clinical benefit is sufficiently demonstrated. The widespread adoption of ultrasound in routine clinical practice represents a notable example of this process [242].

Finally, and perhaps most importantly for routine clinical implementation, novel urinary biomarkers must eventually be incorporated into international clinical guidelines. Endorsement by major nephrology and diabetes societies would increase clinician confidence and facilitate broader adoption in everyday practice. Without overcoming these methodological, economic, and regulatory challenges, the promising role of urinary biomarkers in DKD management may remain only partially realized.

## 9. Conclusions, Translational Challenges and Future Directions

DKD remains a primary contributor to CKD and kidney failure globally. In clinical settings, diagnosis and prognostic evaluation are primarily based on albuminuria and eGFR. While these markers are essential, they capture only a fraction of DKD’s biological complexity and fail to adequately represent its significant clinical and molecular heterogeneity. This limitation is especially pronounced in NADKD, where renal function may deteriorate even when urinary albumin excretion remains within normal limits.

The evidence reviewed in this article demonstrates that emerging biomarkers provide complementary insight into key pathogenic processes involved in DKD, including glomerular permeability, podocyte injury, tubular dysfunction, inflammation, oxidative stress, fibrosis, and endothelial damage. Among the currently available candidates, KIM-1, NGAL, L-FABP, TNFR-1, TNFR-2, and the urinary proteomic classifier CKD273 have shown the most consistent associations with disease progression and adverse renal outcomes across independent cohorts. Importantly, several of these biomarkers appear to provide prognostic information beyond albuminuria and eGFR and may improve risk stratification in patients with NADKD, a phenotype for which conventional diagnostic approaches remain suboptimal. Emerging evidence also supports a role for suPAR as both a biomarker and a potential mediator of DKD progression, while extracellular vesicles represent a promising liquid biopsy platform for future biomarker discovery and molecular characterization.

The application of multi-omics methodologies, including transcriptomics, proteomics, metabolomics, lipidomics, epigenomics, and extracellular vesicle profiling, is significantly advancing the understanding of DKD pathogenesis [284,285,286]. These technologies reveal complex molecular networks underlying disease initiation and progression, moving beyond single pathway, tissue compartment, or clinical phenotype analyses, and increasingly achieving single-cell and spatial resolution. If rigorously validated, multi-omics approaches may facilitate DKD classification that transcends traditional phenotype-based categories, enabling a more precise molecular taxonomy that accurately reflects the disease’s biological heterogeneity [287,288,289,290,291,292,293].

The advancement of DKD biomarker research is unlikely to rely on the identification of a single optimal biomarker. Greater clinical utility is expected from multimarker strategies, increasingly supported by artificial intelligence-based analytical methods, which integrate complementary signals from glomerular, tubular, inflammatory, endothelial, and fibrotic pathways. Currently, TNFR-1, TNFR-2, CKD273, and selected tubular injury biomarkers are closest to clinical application, while extracellular vesicle-derived biomarkers and multi-omics signatures remain promising but require further validation prior to routine use. Transitioning these tools from discovery to clinical practice will necessitate large prospective multicenter studies, assay standardization, validation across diverse populations, health-economic evaluation, and biomarker-guided interventional trials [294,295].

Taken together, these advances represent an important turning point in the evolving approach to DKD detection, risk prediction, and management. Nevertheless, the gap between biomarker promise and clinical implementation remains substantial. Molecular profiling, advanced biomarker panels, and data-driven prediction models may ultimately enable earlier diagnosis, more accurate prognostic assessment, and truly individualized therapeutic strategies. At present, however, none has been sufficiently standardized or validated, or shown to improve patient outcomes, to justify widespread incorporation into routine clinical care. The central challenge for the field is therefore no longer simply to identify additional candidate biomarkers, but to demonstrate through rigorous validation and biomarker-guided clinical studies that the most promising candidates can improve patient outcomes and advance precision medicine in DKD.

## Data Availability

No new data were created or analyzed in this study. Data sharing is not applicable to this article.
